# Integrated Molecular Characterization of Patient-Derived Models Reveals Therapeutic Strategies for Treating CIC-DUX4 Sarcoma

**DOI:** 10.1158/0008-5472.CAN-21-1222

**Published:** 2021-12-13

**Authors:** Marianna Carrabotta, Maria Antonella Laginestra, Giorgio Durante, Caterina Mancarella, Lorena Landuzzi, Alessandro Parra, Francesca Ruzzi, Lisa Toracchio, Alessandra De Feo, Veronica Giusti, Michela Pasello, Alberto Righi, Pier-Luigi Lollini, Emanuela Palmerini, Davide Maria Donati, Maria Cristina Manara, Katia Scotlandi

**Affiliations:** 1 Experimental Oncology Laboratory, IRCCS Istituto Ortopedico Rizzoli, Bologna, Italy.; 2 Laboratory of Immunology and Biology of Metastasis, Department of Experimental, Diagnostic and Specialty Medicine (DIMES), University of Bologna, Bologna, Italy.; 3 Department of Pathology, IRCCS Istituto Ortopedico Rizzoli, Bologna, Italy.; 4 Osteoncology, Bone and Soft Tissue Sarcoma and Novel Therapy Unit, IRCCS Istituto Ortopedico Rizzoli, Bologna, Italy.; 5 Third Orthopaedic Clinic and Traumatology, IRCCS Istituto Ortopedico Rizzoli, Bologna, Italy.; 6 Department of Biomedical and Neuromotor Sciences (DIBINEM), University of Bologna, Bologna, Italy.

## Abstract

Capicua-double homeobox 4 (CIC-DUX4)–rearranged sarcomas (CDS) are extremely rare, highly aggressive primary sarcomas that represent a major therapeutic challenge. Patients are treated according to Ewing sarcoma protocols, but CDS-specific therapies are strongly needed. In this study, RNA sequencing was performed on patient samples to identify a selective signature that differentiates CDS from Ewing sarcoma and other fusion-driven sarcomas. This signature was used to validate the representativeness of newly generated CDS experimental models—patient-derived xenografts (PDX) and PDX-derived cell lines—and to identify specific therapeutic vulnerabilities. Annotation analysis of differentially expressed genes and molecular gene validation highlighted an HMGA2/IGF2BP/IGF2/IGF1R/AKT/mTOR axis that characterizes CDS and renders the tumors particularly sensitive to combined treatments with trabectedin and PI3K/mTOR inhibitors. Trabectedin inhibited IGF2BP/IGF2/IGF1R activity, but dual inhibition of the PI3K and mTOR pathways was required to completely dampen downstream signaling mediators. Proof-of-principle efficacy for the combination of the dual AKT/mTOR inhibitor NVP-BEZ235 (dactolisib) with trabectedin was obtained *in vitro* and *in vivo* using CDS PDX-derived cell lines, demonstrating a strong inhibition of local tumor growth and multiorgan metastasis. Overall, the development of representative experimental models (PDXs and PDX-derived cell lines) has helped to identify the unique sensitivity of the CDS to AKT/mTOR inhibitors and trabectedin, revealing a mechanism-based therapeutic strategy to fight this lethal cancer.

**Significance:**

This study identifies altered HMGA2/IGF2BP/IGF2 signaling in CIC-DUX4 sarcomas and provides proof of principle for combination therapy with trabectedin and AKT/mTOR dual inhibitors to specifically combat the disease.

## Abstract (precis)

This study identifies altered HMGA2/IGF2BP/IGF2 signaling in CIC-DUX4 sarcomas and provides proof of principle for combination therapy with trabectedin and AKT/mTOR dual inhibitors to specifically combat the disease.

## Graphical Abstract

**Figure:**
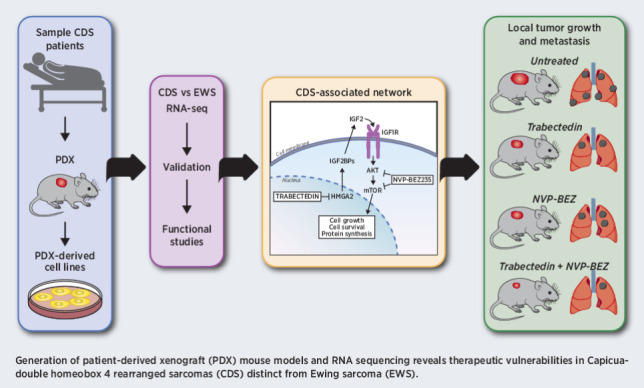

## Introduction

Sarcomas are a group of tumors with numerous subtypes that exhibit unique clinical and prognostic characteristics. The fifth edition of the World Health Organization (WHO) Classification of Tumors of Soft Tissue and Bone recognizes this heterogeneity and accounts for advances in diagnosing sarcoma subtypes, largely due to the advent of next-generation sequencing techniques that allow the detection of novel gene fusions (1). This scenario is particularly true for small round blue cell tumors, for which several novel molecular subtypes have been defined in recent years (2). Capicua–double homeobox 4 (CIC-DUX4) rearranged sarcoma (CDS) is a subcategory of small round blue cell tumors defined by the presence of the oncogenic driver CIC-DUX4 hybrid protein. CDS resembles the morphologic phenotypes of Ewing sarcoma (EWS), were originally defined as Ewing-like tumors (3) and treated with the same therapeutic regimens of Ewing sarcoma. However, recent clinicopathologic and molecular genetic analyses have indicated that CDS is an independent disease entity (4). While Ewing sarcoma is a prevalent pediatric bone tumor, highly chemosensitive and with a good prognosis if localized at diagnosis (5), tumors with CIC-DUX4 fusions appear in children, adolescents, and adults (range, 15–44 years; mean, 32 years), usually arise in soft tissues with only rare osseous involvement (6), show a high metastatic rate and quickly develop resistance to chemotherapy (7–9). Overall, patients with CDS have a substantially, less favorable outcome than those with Ewing sarcoma; the median survival is less than 2 years, and effective therapeutic strategies for CDS are urgently required. In this study, we used RNA sequencing (RNA-seq) to reveal distinct transcriptomic patterns and new therapeutic opportunities to specifically combat CDS.

The genetic rearrangement that leads to the *CIC-DUX4* fusion gene typically connects the *Capicua* (*CIC*) gene (19q13) to *DUX4* (4q35 or 10q26; ref. 8). *CIC* is an evolutionarily conserved transcription factor containing a high-mobility group (HMG) box that recognizes specific DNA sequences. CIC acts as a transcriptional repressor to regulate receptor tyrosine kinase signaling pathways, particularly MAPK/ERK, and thereby controls several developmental and physiologic processes (10, 11). *DUX4* is a double-homeobox gene that belongs to a family of double homeodomain transcriptional activators, which are normally expressed in human embryos before they are epigenetically silenced for the rest of development and throughout a person's life. The CIC-DUX4 fusion protein generally promotes the expression of downstream targets, such as *ETS Variant Transcription (ETV) Factors and G1/S-specific cyclin D (CCND)2* (9). However, the molecular targets regulated by the CIC-DUX4 fusion protein that promote the molecular pathogenesis of CDS remain largely unknown.

By transducing embryonic mesenchymal cells or NIH 3T3 mouse fibroblasts with human *CIC-DUX4* cDNA, researchers have identified gene expression profiles that characterize CDS (9, 12). Here, we went one step further in identifying the specific genetic profile that differentiates human CDS from Ewing sarcoma and the other fusion-driven sarcomas by using RNA-seq. We uncovered a molecular dependence of CDS tumors on an oncogenic signaling pathway that involves HMGA proteins and insulin-like growth factor 2 binding protein (IGF2BP)2 and IGF2BP3. HMGA2, a chromatin modifier, was shown to activate the transcription of *IGF2BP2* and/or *IGF2BP3* (13–15), which in turn bind to and control the translation of a set of mRNAs, including *IGF2* and *IGF1R* with the subsequent autocrine activation of downstream pathways (16, 17). CDS patient-derived xenograft (PDX) and PDX-derived cell lines were exploited to identify specific therapeutic vulnerabilities of CDS. In particular, this study provides proof of concept for the efficacy of trabectedin, which is known to displace HMGA proteins from HMGA-responsive promoters (18), in combination with anti-AKT/mTOR-targeted agents and helps to define specific therapeutic approaches for patients with CDS.

## Materials and Methods

### RNA-seq library preparation

Four soft tissue CDS and four soft tissue Ewing sarcoma frozen samples (obtained from the Musculoskeletal Tumor Biobank of the Rizzoli Institute, Bologna, Italy), were evaluated by a pathologist who certified the high-density cancer areas (>70%) and processed for RNA extraction.

Total RNA from patient samples, PDXs and PDX-derived cell lines was isolated using TRIzol Reagent (#15596026, Thermo Fisher Scientific). RNA quality was assessed using an Agilent Bioanalyzer (version 2100; RRID:SCR018043) to obtain a RNA integrity number higher than seven.

For each sample, 800 ng of total RNA was used to synthesize cDNA libraries using the TruSeq Stranded Total RNA Kit with RiboZero Gold (20020599, Illumina) according to the manufacturer's recommendations. The libraries were paired end sequenced (2 × 75 bp) on an Illumina NextSeq 500 (RRID:SCR014983) following the manufacturer's instructions, generating an average of approximately 54 million 75 bp paired end raw reads per sample.

### RNA-seq data analysis

FASTQ files were analyzed using RNA Express BaseSpace App (Illumina, RRID:SCR_011881). Raw counts were normalized according to the library size to obtain transcripts per million and 14,334 genes were used to generate unsupervised hierarchical clustering (HC) and three-dimnsional (3D) principal component analysis (PCA). Unsupervised HC was generated using the Hclust R function (RRID:SCR_009154) based on the Ward.D2 method and Euclidean distance as a measure of similarity (R package stats v3.6.2). PCA was conducted using the pricomp R function (19) and visualized using the 3D visualization device system RGL (R package version 0.100.54; https://CRAN.R-project.org/package=rgl/). Starting from the DeSeq2 output, we performed several steps of data selection and filtration. The list of these genes was additionally filtered for a Benjamini-Hochberg (BH)-adjusted *P* < 0.05; we retained the genes in which the single gene level counts in the two groups (Ewing sarcoma and CDS) had a mean raw count >5 for each group. A coefficient of variation (CV) analysis for the filtered genes was performed in the two sample groups (CDS and Ewing sarcoma) to evaluate the level of expression value dispersion around the mean.

The selected signature was used for Spearman correlation analysis among patients with CDS, PDXs and corresponding PDX-derived cell lines. The confidence interval (95% confidence level) for each correlation coefficient was performed. In addition, we used statistical significance tests for comparing intragroup and intergroup correlation coefficients.

Heatmaps and correlograms were generated using the Complex Heatmap (RRID:SCR017270) and CorrPlot R packages, respectively (20). All analyses were performed using R version 3.6 (Bioconductor, http://www.bioconductor.org/; RRID:SCR_006442).

Functional analysis was performed using gene set enrichment analysis (GSEA). The Molecular Signature Database (MsigDB) c2.all.v7.0.symbols.gmt signature (RRID:SCR_016863) was used and gene sets with between 15 and 200 members were considered (21). The most relevant gene sets were selected considering a normalized enrichment score ≥2.5 and a FDR ≤ 0.001. In addition, a leading edge analysis was performed to investigate key genes related to the transcriptomic changes of CDS samples (21).

To validate the gene signature/s characteristic of CDS, we considered a published microarray-based gene expression dataset of 14 CDS, seven EWSR1-NFATc2 (GSE60740), 27 Ewing sarcoma (E-MEXP-1142), eight monophasic synovial sarcomas (MSS), 6 myxoid liposarcomas (MLS), and 4 rhabdomyosarcoma (ARMS) (E-MEXP-353) patient-derived tumors and 17 normal mesenchymal stem cells (MSC; GSE7888). Raw CEL file data were normalized using Robust Multi-Array Average normalization and log_2_ transformed. To reduce the variability across different datasets, we performed batch effect correction using the removeBatchEffect function from the limma R package Unsupervised HC was adopted to test the gene signature, and Z-scores of log_2_-transformed expression values were displayed using the ComplexHeatmap R package (RRID:SCR017270; ref. 20).

The web-based software MetaCore (GeneGo, Thomson Reuters, RRID:SCR008125) was used to create interaction networks for the genes resulting from leading edge analysis.

Detailed information is provided in the Supplementary Data.

### Establishment of CDS PDXs and PDX-derived cell lines

To generate PDXs, a fresh tumor specimen approximately 4 mm^3^ in volume was implanted subcutaneously at the level of interscapular brown fat into 5–11 weeks old immunodeficient NOD scid gamma (NSG) mice (Charles River) as described previously (22). Additional details are included in Supplementary Data.

### Histopathology and IHC

Serial 3-μm-thick paraffin sections from original tumors and PDXs were processed according to standardized automated procedures (Ventana Medical Systems) and then immunostained with the following antibodies: CD99 (Ventana, mouse monoclonal antibody O13, prediluted), ETV4 (Santa Cruz Biotechnology, clone PEA3, 1/20), or buffer alone (negative control); p-AKT (Ser473; 736E11, rabbit, #3787, 1:50, Cell Signaling Technology, RRID:AB_331170); phospho-mTOR (Ser2448; polyclonal, rabbit, #2971S, 1:50, Cell Signaling Technology, RRID:AB_330970); phospho-S6 ribosomal protein (Ser240/244; rabbit, #2215, 1:30, Cell Signaling Technology, RRID:AB_2238583). For morphologic analyses, the slides were stained with hematoxylin/eosin.

### Preclinical studies

The cell lines PDX-CDS#1-C, PDX-CDS#3-C, and PDX-CDS#4-C were obtained from CDS PDXs after 1–3 passages in the animal. The cell lines PDX-EWS#2-C, PDX-EWS#4-C, and PDX-EWS#5-C were obtained from the corresponding Ewing sarcoma PDXs after the first passage in mice (22). All cell lines were authenticated by DNA fingerprinting using POWERPLEX ESX 17 Fast System, (#DC1710, Promega). The details of all cell culture condition, quality control assays, and *in vitro* drug treatments are reported in the Supplementary Data. Quantitative real-time (qRT)-PCR analysis was performed using a ViiA 7 Real-Time PCR System (Thermo Fisher Scientific, RRID:SCR019582) under standard conditions. In each experiment, samples were run in duplicate. Relative quantification analysis was performed using the 2^–ΔΔ*C*_t_^ method (23). Primer sequences are reported in the Supplementary Data. Western blotting was executed according to standard procedures. Proteins of interest were detected using specific antibodies, with additional details provided in Supplementary Data.

### Chemicals

Doxorubicin hydrochloride (#D1515), vincristine, and insulin growth factor 2 (IGF2, #I2526) were purchased from Merck. Irinotecan (#S2217), MK-2206, capivasertib (AZD5363), alpelisib (HY-15244), nilotinib (S1033), pazopanib (S3012), and ponatinib (S1490) were purchased from Selleckchem. Everolimus (RAD001, SRP020750e) was purchased from Sequoia Research Products, etoposide was purchased from Sandoz and AVE 1642 from Immunogen. D-188514, an ifosfamide analog that does not require metabolic activation, was purchased from Niomech. The PI3K/mTOR dual inhibitor NVP-BEZ235 was kindly provided by Novartis. Trabectedin (ET-743) was kindly provided by PharmaMar. Working dilutions of all drugs were prepared immediately before use.

### Pharmacologic *in vivo* experiments

BALB/c Rag2^−/−^;Il2rg^−/−^ breeders were kindly provided by the Central Institute for Experimental Animals (24). The mice were bred in the Animal Care Facility of the Laboratory of Immunology and Biology of Metastasis, (Pier-Luigi Lollini). PDX-CDS#4-C (2 × 10^6^ cells) were injected subcutaneously to assess tumor growth or intravenously to assess experimental metastasis. The animals were randomized into controls and treatment groups to evaluate the drug efficacy of trabectedin, NVP-BEZ235 or their combination. Treatments started when tumor volumes were measurable (5 mm^3^) or 7 days after intravenous cell injection. Details are provided in the Supplementary Data.

### Statistical analysis

GraphPad Prism (version 7.0 Software; RRID:SCR002798) was employed to perform statistical analysis. Differences among the means were analyzed using Student *t* test or one-way ANOVA when the experimental data included more than two groups. IC_50_ values were calculated from linear transformation of dose–response curves using CalcuSyn software version 2 (Biosoft; RRID:SCR_020251). The combination index (CI) was calculated with an isobologram equation using CalcuSyn software to identify drug–drug effects according to Chou and colleagues (25). Tumor-free survival curves were computed using the Mantel–Cox log-rank test and a *P* ≤ 0.05. Differences in metastasis number were analyzed using the nonparametric Mann–Whitney test.

### Study approval

The study was approved by the ethics committee of the IRCCS Istituto Ortopedico Rizzoli (Prot.Gen 0009323 2016/04/22, Prot.Gen 0009164 2017/09/22, Prot. Gen 0011371 2019/09/25). Patient-informed consent forms were obtained for biobanking and establishment of PDX models; all methods were performed in accordance with institutional guidelines and Italian law. All animal procedures were performed in accordance with ARRIVE guidelines (26), European directive 2010/63/UE and Italian Law (DL 26/2014); experimental protocols were approved by the institutional animal care and use committee of the University of Bologna and by the Italian Ministry of Health (authorizations 782/2015-PR, 208/2017-PR, and 755/2018-PR).

### Data and materials availability

All sequencing data that support the findings of this study have been deposited in the National Center for Biotechnology Information Gene Expression Omnibus (GEO) and are accessible through the GEO Series accession number GSE165032. PDXs and PDX-derived cell lines, not available through public repositories, are available from the corresponding authors on request under a material transfer agreement with the IRCCS Istituto Ortopedico Rizzoli. A virtual machine reproducing the full analysis environment is available on Code Ocean (https://codeocean.com/capsule/7346072/tree/v1). All other data are present in the main text or in the Supplementary Data.



## Results

### RNA-seq analysis of CDS reveals a distinct transcriptional profile from Ewing sarcoma and highlights genes and networks specific to CDS

To investigate the molecular signature of CDS, we used RNA-seq and performed unsupervised HC to detect differences in gene expression profiles between CDS and Ewing sarcoma patient tumors (Supplementary Fig. S1A). Because we were aware of the low number of patients here examined, we tried to avoid other limitations and we selected soft tissue Ewing sarcoma for the comparison with soft tissue CDS. The distinctive transcriptional profile of CDS compared with Ewing sarcoma was further explored using 3D PCA, which showed that CDS cases formed a cloud distinct from the Ewing sarcoma cases (Supplementary Fig. S1B).

On the basis of a BH *P*_adj_ < 0.05, we identified 3,179 deregulated genes (DEG) (1,458 downregulated and 1,721 upregulated) in CDS compared with patients with Ewing sarcoma (Supplementary Fig. S1C). Applying a more restrictive threshold (BH *P*_adj_ < 0.01 and absolute FC ≥ 2.5) we obtained a list of 537 DEGs (275 upregulated and 262 downregulated genes) in CDS compared with Ewing sarcoma patient tumors (Supplementary Table S1). As expected, *ETV4* and *ETV1* were among the most upregulated genes (Supplementary Fig. S1C), confirming their specific and sensitive transcriptional regulation as downstream targets of the *CIC-DUX4* fusion gene (12, 27, 28).

**Figure 1.**
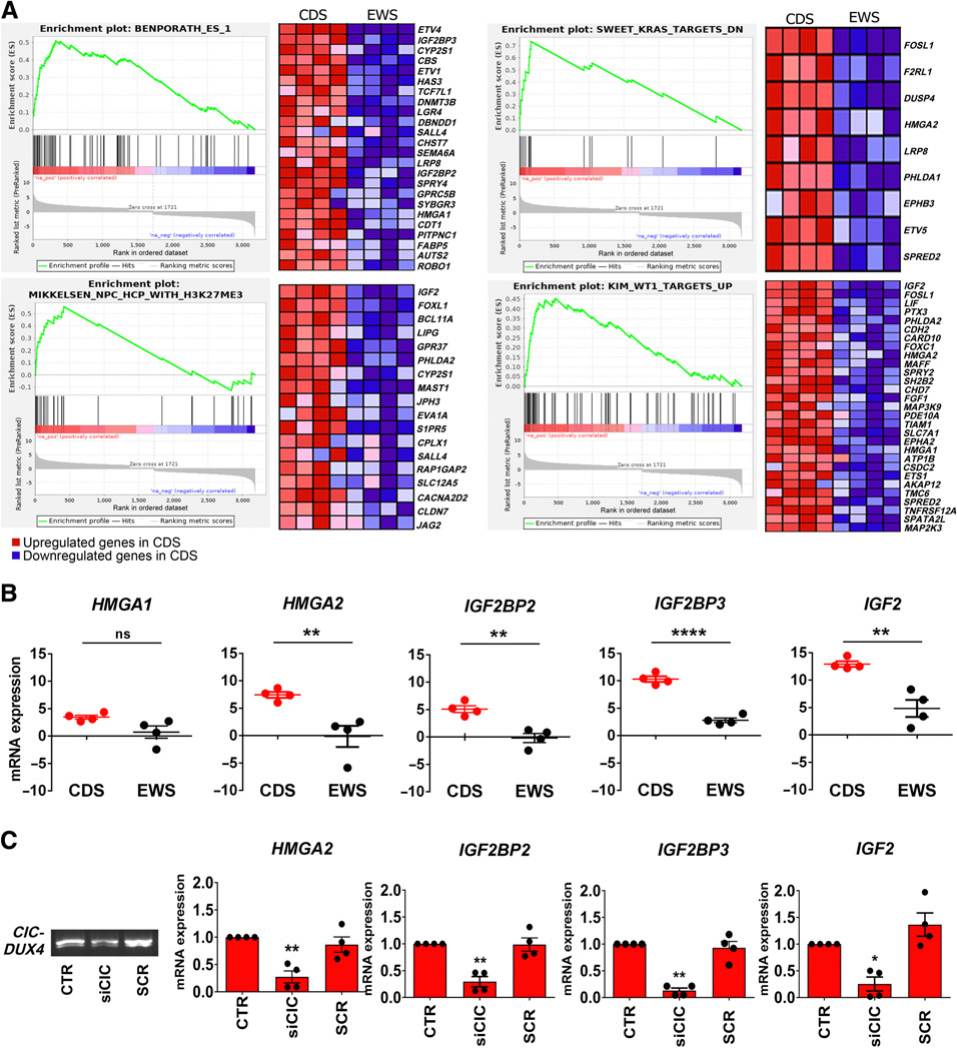
GSEAs and qRT-PCR validation reveal a unique transcriptional profile of CDS characterized by enriched expression of a *HGMAs/IGF2BPs/IGF2* axis. **A,** GSEA reveals a significant enrichment of genes upregulated in patients with CDS versus Ewing sarcoma. CDS displayed a signature enriched for genes involved in embryonic stem cell development (Benporath_ES_1), *KRAS* signaling (Sweet_KRAS_TARGETS_DN), *WT1* target genes (Kim_WT1_TARGETS_UP), and chromatin modification (Mikkelsen_NPC_HCP_WITH_H3K27ME3). The enrichment score curve was obtained using GSEA software. In the enrichment plot, the *x*-axis shows the rank order of genes from the most upregulated to the most downregulated between CDS and Ewing sarcoma samples. The vertical black line indicates the position of the enriched genes (Hit) comprising the gene set. The graph on the bottom shows the ranked list metric (signal-to-noise ratio) for each gene as a function of the rank in the ordered dataset. The heatmaps show the genes that contribute most to the enriched pathway or biological process resulting from the leading edge analysis. **B,** Validation of the RNA-seq results was performed by qRT-PCR and the results are shown in scatter plots. Each dot represents a tumor sample. Differential expression of genes between CDS and Ewing sarcoma was established by Student *t* test and correction for multiple comparisons using the Holm-Sidak method: **, *P* < 0.01; ****, *P* < 0.0001; ns, nonsignificant. The mean ± SE of relative mRNA expression (2^–ΔΔ*C*_t_^) is reported as log_2_. *GAPDH* was used as a reference gene. **C,** Left, *CIC-DUX4* silencing in PDX-CDS#4 cells by RT-PCR. Agarose gel electrophoresis image of a 233-base fusion transcript is shown. Right, relative mRNA expression (2^−ΔΔ*C*_t_^) of *HMGA2*, *IGF2BP2*, *IGF2BP3*, and *IGF2* after cell exposure to siCIC or scrambled (SCR) control siRNAs (40 nmol, 72 hours) by qRT-PCR. The mean ± SE of relative mRNA expression (2^−ΔΔ*C*_t_^) is reported as log_2_. *GAPDH* was used as a reference gene. *, *P* < 0.05; **, *P* < 0.01; one-way ANOVA with respect to the control.

To reveal novel classes of genes that are specifically overrepresented in CDS, we next focused on identifying biological functions/networks with discriminatory and putative therapeutic value. GSEA was performed using GSEA-Preranked against our ranked list of 3,179 DEGs. Considering a NES ≥2.5 and an FDR ≤ 0.001, we identified 19 top gene sets that were selectively enriched in the CDS (Supplementary Table S2) and performed a leading edge analysis comparing the 19 gene sets with recognize the subset of genes that contributed the most to the core enrichment. We identified 71 enriched genes that were associated with four gene sets: embryonic stem cell-like phenotypes (29), histone H3 trimethylation mark at K27 in neural progenitor cells (NPC; ref. 30), potential effectors of oncogenic *KRAS2* (31) and *Wilms' tumor* (*WT*)*1* signaling transcriptional regulation of the EGF family of growth factors (Fig. 1A; Supplementary Table S3; ref. 32). On the other hand, the GSEA results confirmed that the CDS transcriptional profile was negatively correlated with the gene signature previously reported by Riggi and colleagues (Supplementary Fig. S2; ref. 33), which specifically identified downstream targets of Ewing sarcoma-Friend leukemia integration (FLI; a fusion protein associated with Ewing sarcoma).

To strengthen the value and specificity of these genetic signatures, we used publicly available data from other human fusion-driven sarcomas, and normal mesenchymal stem cells as validation step. Unsupervised HC algorithm confirmed that the 3,179 DEGs and the 537 DEGs signatures were able to correctly distinguish MSCs from other closely related fusion sarcomas, and to distinguish CDSs from EWSR1-NFATc2, Ewing sarcoma, MSS, MLS, and ARMS (Supplementary Figs. S3 and S4). Even when the 71 most restrictive gene signature was tested, we observed a distinct cluster of CDS from other sarcoma tumors and mesenchymal stem cells (Supplementary Fig. S5), further supporting the specificity of our results. Together, these results confirm that the three gene signatures were able to delineate homogeneous groups of tumors and to define a distinct transcriptomic pattern of CDSs. In addition, we specifically analyzed the selected genes in the context of their expression performing a CV analysis for the 3,179 genes in both the two groups (CDS and Ewing sarcoma; CV threshold ≤0.25). Only 13 genes in CDS and 33 genes in Ewing sarcoma of the 3,179 genes had a CV equal to or above 0.25 (Supplementary Fig. S6). None of these genes were in the more restricted list (71 genes) that was considered for all the downstream functional analysis.

Using Metacore software, we then evaluated how the 71 genes are mutually interconnected and identified three main networks (Supplementary Fig. S7) as major regulators of functional interactions: one involving IGF2-IGF2BP2/IGF2BP3 and HMGA1/2; a second starting from G protein-coupled receptors and ephrin receptors focused on the transcription factors ETS1, Transcription Factor 7 Like (TCF7L)1 and ETV1/4/5; and a third starting from Claudin-7 and Roundabout Guidance Receptor (ROBO)1 that leads to *Forkhead Box L* (*FOXL*)*1* as major regulator of functional interactions. Because previous work found that HMGAs are modulated after transducing mesenchymal cells with human CIC-DUX4 (9, 12), and that both HGMAs and IGF2BPs are specifically expressed in CIC-fused compared with other fusion-driven sarcomas (34), we focused further analysis on the relationship that links HMGAs to IGF2BP2/IGF2BP3 and to IGF2. qRT-PCR confirmed that CDS specimens exhibited significantly elevated expression of *HMGA2*, *IGF2BP2*, *IGF2BP3*, and *IGF2*, but not *HMGA1*, compared with Ewing sarcoma specimens (Fig. 1B). Targeting of the *CIC-DUX4* fusion transcript by CIC RNA interference abrogates the expression of *HMGA2*, *IGF2BP2*, *IGF2BP3*, and *IGF2* in siRNA-treated cells compared with controls at the mRNA level further sustaining the specificity of our findings (Fig. 1C).

### PDXs and PDX-derived cell lines maintain the phenotypic characteristics and transcriptional profiles of primary CDS tumors

**Figure 2.**
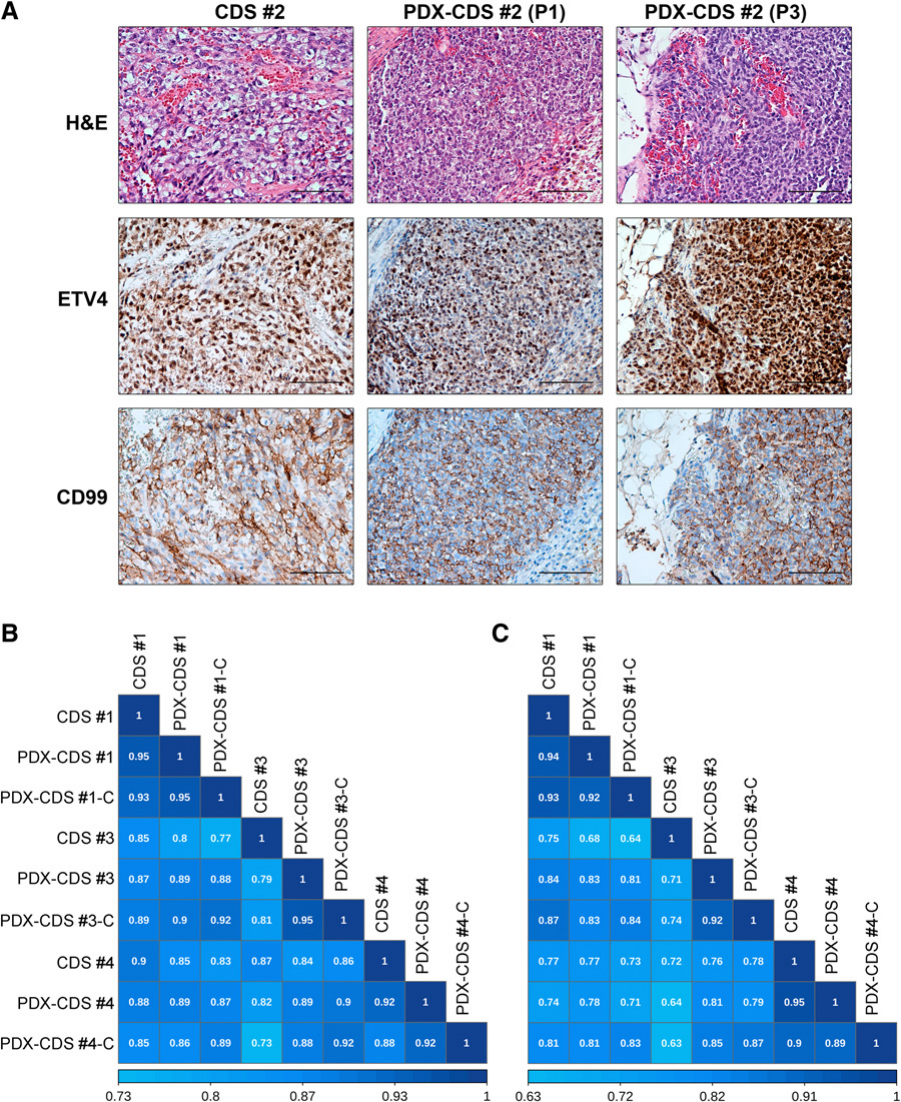
CDS PDX models recapitulated the phenotypic characteristics and transcriptional profiles of the primary tumor. **A,** Histologic and IHC features of CDS patient samples and corresponding PDXs at different *in vivo* passages (P1, first passage; P3, third passage). Sections were stained with hematoxylin-eosin (H&E) or with antibodies against the antigens ETV4 and CD99. Bar, 100 μm. **B** and **C,** Spearman correlation analysis among the gene expression profiles of CDS tumors, their corresponding PDX models (P3), and the PDX-derived cell lines are shown based on the signatures of 3,179 (**B**) and 71 (**C**) DEGs. Correlation coefficients are shown.

PDXs are an invaluable tool for understanding tumorigenesis and developing novel therapeutic strategies; unfortunately, there are very few available PDXs of primary CDS (35). Here, we successfully established PDXs from the previously characterized CDS and subsequently generated CDS cell lines from grafted tumors. PDX minimal information is summarized in Supplementary Table S4 (36). The success rate for the engraftment of CDS PDXs was 100% (4/4), compared with 24% for Ewing sarcoma PDXs (22), which is in line with the more aggressive nature of this disease. The median latency of CDS PDXs was 6 weeks (range, 2–30 weeks) in the first generation, 4.5 weeks (range, 3–13 weeks) in the second generation, and 3 weeks (range, 1–9) in the third generation, suggesting a progressive selection for the most aggressive cells in each generation. Metastatic spontaneous dissemination to the lung was observed in two of four CDS PDXs, (PDX-CDS#4: incidence 50%; range, 0–4; PDX-CDS#1: incidence 13%; range, 1–0). For comparison, the median latency for Ewing sarcoma PDXs was 27 weeks in the first generation and 4 weeks in the fourth generation (22), while metastatic growth in Ewing sarcoma PDXs was reported only for PDX-EWS#4 (incidence 14%; range, 0–3; ref. 22). CDS xenografts up to the third generation were histologically similar between the original patient tumors (Fig. 2A; Supplementary Fig. S8), and the expression of typical biomarkers, such as ETV4, was confirmed. Even the expression patterns of antigens that are found only in a minority of CDS cells, such as cluster of differentiation (CD)99, were consistent with those of the original patient tumors and the corresponding PDXs (Fig. 2A; Supplementary Fig. S8). Tumor cells were recovered from the xenografts and seeded onto culture dishes to generate PDX-derived cell lines; three of four cases had successful cell line generation. The presence of the *CIC-DUX4* rearrangement was confirmed by RT-PCR in all three PDX-derived cell lines (named PDX-CDS#1-C; PDX-CDS#3-C and PDX-CDS#4-C) as well as in the original tumors and corresponding PDXs (Supplementary Fig. S9).

Then, to assess genetic similarities between PDXs, PDX-derived cell lines, and the original human tumors, we performed Spearman correlation analysis considering either the 3,179 DEGs (range rho = 0.73–0.95) or the 71 DEG signature (range rho = 0.63–0.95; Fig. 2B and C; confidence interval (95% confidence level) are reported in Supplementary Tables S5 and S6). Because some correlation coefficients across samples derived from different tumors are higher than correlations between samples from the same tumor, we performed a comparison between the correlation coefficients to assess the statistical differences within or among the CDS/PDX/PDX-derived cell lines models (intragroup analysis; Supplementary Table S7; intergroup analysis Supplementary Table S8). The intragroup analysis highlighted that PDX-CDS#3 and PDX-CDS#3-C showed statistical differences (Bonferroni *P*_adj_ value) with respect to the original patient when we considered the 3,179 DEGs but not when the restricted 71 DEG signature, indicating that also these two models maintain a good representativeness of the patient's tumor for the genes that we focused on.

Overall, PDXs and PDX-derived cell lines replicate the most characterizing morphologic and genetic features of patients with CDS, supporting the usefulness of these models for preclinical studies.

### Effective therapeutic approaches for CDS treatment

**Figure 3.**
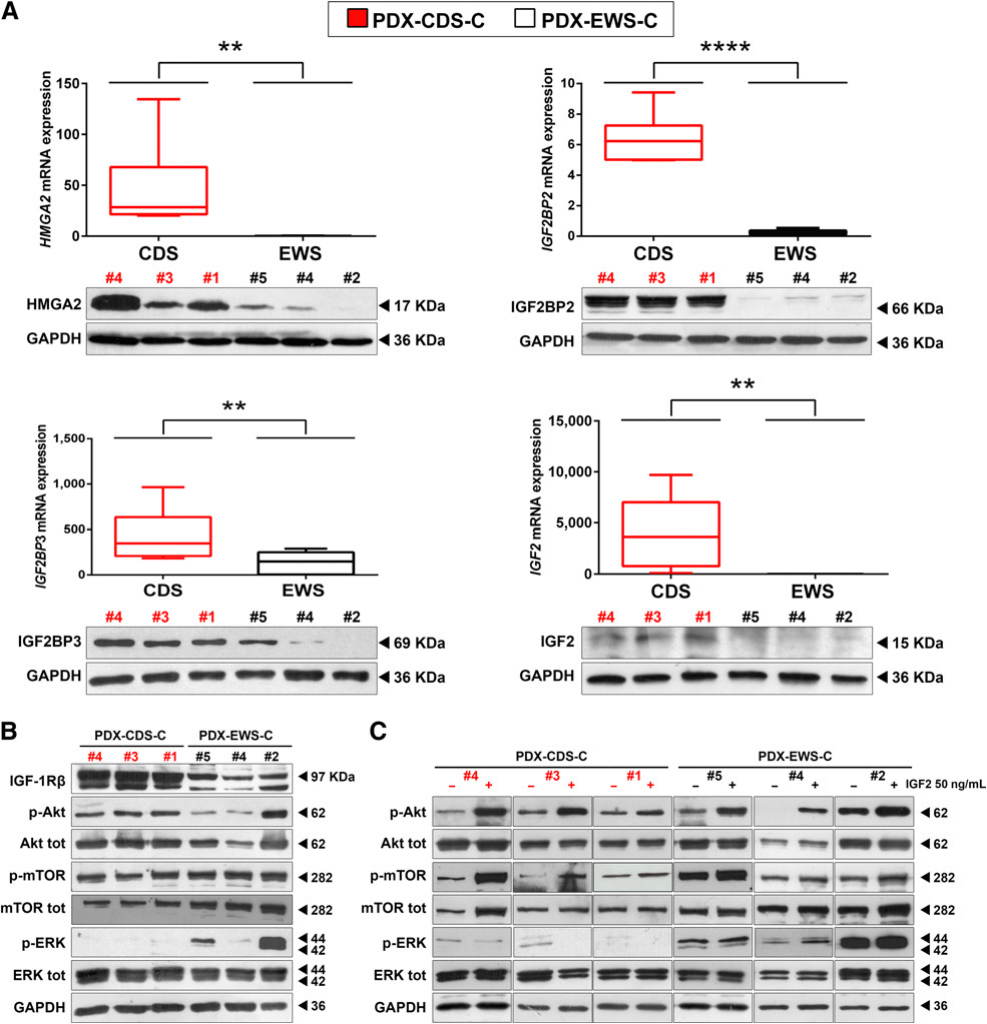
The HMGA2/IGF2BP2–3/IGF2 network is upregulated and sustains AKT pathway activity in CDS PDX-derived cell lines versus Ewing sarcoma PDX-derived cell lines. **A,** qRT-PCR (top) and Western blot (bottom) analysis of *HMGA2*, *IGF2BP2*, *IGF2BP3*, and *IGF2* mRNA and protein in CDS PDX-derived cell lines versus Ewing sarcoma PDX-derived cell lines. Three independent biological replicates were performed for qRT-PCR and Western blotting. One representative immunoblot is shown. For qRT-PCR, the data represent the mean ± SE. **, *P* < 0.01; ****, *P* < 0.0001, Student *t* test comparing the CDS and Ewing sarcoma groups. *GAPDH* was used as a reference gene. **B,** Immunoblots showing the protein expression of IGF1R and the downstream mediators AKT and MAPK under basal conditions. Two independent experiments were performed. One representative immunoblot is shown. GAPDH was employed for normalization purposes. **C,** Immunoblots showing the expression of AKT and MAPK after serum starvation with or without subsequent stimulation with IGF2. Two independent experiments were performed. One representative immunoblot is shown. GAPDH was employed for normalization purposes.

In keeping with the hypothesis that we can identify druggable vulnerabilities useful for the treatment of CDS by revealing genes specifically upregulated in CDS versus Ewing sarcoma, we checked the expression of mRNA and proteins in the HMGA2/IGF2BPs/IGF2 axis in CDS and Ewing sarcoma PDX-derived cell lines. *HMGA2*, *IGF2BP2*, *IGF2BP3*, and *IGF2* were confirmed to be significantly overexpressed in CDS-derived cell lines compared with Ewing sarcoma–derived cell lines (Fig. 3A). Data from the literature indicate that HMGA2 promotes the transcription of *IGF2BP2* (37), while IGF2BP2 and IGF2BP3 directly bind to and stabilize *IGF2* and *insulin-like growth factor 1 receptor (IGF1R)* mRNAs (38). Indeed, silencing *HGMA2* by RNAi in PDX-CDS cells led to decreased expression of IGF2BP2/IGF2BP3 at both RNA and protein level (Supplementary Fig. S10) confirming the existence of HMGA2-driven signaling. Accordingly, under basal conditions, CDS PDX-derived cell lines displayed higher constitutive expression of IGF1R and increased activation of the AKT pathway than did Ewing sarcoma PDX-derived cell lines (Fig. 3B).

Interestingly, ERK activity was severely dampened in CDS cells, likely because of its interaction with and subsequent repression by CIC-DUX4 (10). Indeed, stimulation of serum-starved PDX-CDS or PDX-Ewing sarcoma cells with IGF2 led to ERK activation in Ewing sarcoma cells but not in CDS cells, while AKT activation was observed in both CDS and Ewing sarcoma cells (Fig. 3C). When CDS cells were exposed to the neutralizing anti-IGF1R hAb AVE 1642 (Supplementary Fig. S11; ref. 39), AKT signaling was disrupted, confirming that the activation of IGF1R by autocrine production of its ligand leads to functions that are mainly driven by the AKT pathway in CDS cells. Consequently, we tested the sensitivity of CDS cells to several tyrosine kinase inhibitors (Table 1). CDS cells were found to be more sensitive to the dual PI3K/mTOR inhibitor NVP-BEZ235 (40), which has been reported as a promising candidate for the treatment of sarcoma (40, 41), than to other selective PI3K, AKT, and mTOR inhibitors (such as alpelisib, MK-2206, capivasertib and everolimus) or multi-kinase inhibitors (such as pazopanib, ponatinib, nilotinib). Combined simultaneous treatment with alpelisib (PI3K inhibitor) and everolimus (mTOR inhibitor) led to additive/synergistic effects, confirming the need for dual inhibition (Table 1).

**Table 1.** Drug sensitivity in CDS and Ewing sarcoma PDX-derived cell lines.

	PDX-CDS #1-C^a^	PDX-CDS #3-C^a^	PDX-CDS #4-C^a^
NVP-BEZ235 (μmol/L)	2.5 ± 0.3	0.15 ± 0.1	0.09 ± 0.03
MK-2206 (μmol/L)	>30	5.8 ± 2.1	6.1 ± 1.3
Capivasertib (μmol/L)	>30	7.3 ± 0.4	9.9 ± 4.4
Pazopanib^b^ (μmol/L)	>30	>30	>30
Ponatinib^b^ (μmol/L)	>30	>30	>30
Nilotinib^b^ (μmol/L)	>30	>30	>30
Alpelisib^a^ (μmol/L)	26.1 ± 0.3	1.3 ± 0.3	20.8 ± 7.1
Everolimus^b^ (μmol/L)	>30	>30	>30
Alpelisib + Everolimus (μmol/L)	11.8 ± 2.2^c^	0.5 ± 0.1^c^	0.9 ± 0.5^d^

Note: IC_50_ values after 72 hours of treatment are reported.

^a^FDA approved.

^b^FDA and EMA approved.

^c^CI indicates additive (0.9 ≤ CI ≤ 1.1) or ^d^synergistic (CI < 0.9) effects with respect to single agents.

**Figure 4.**
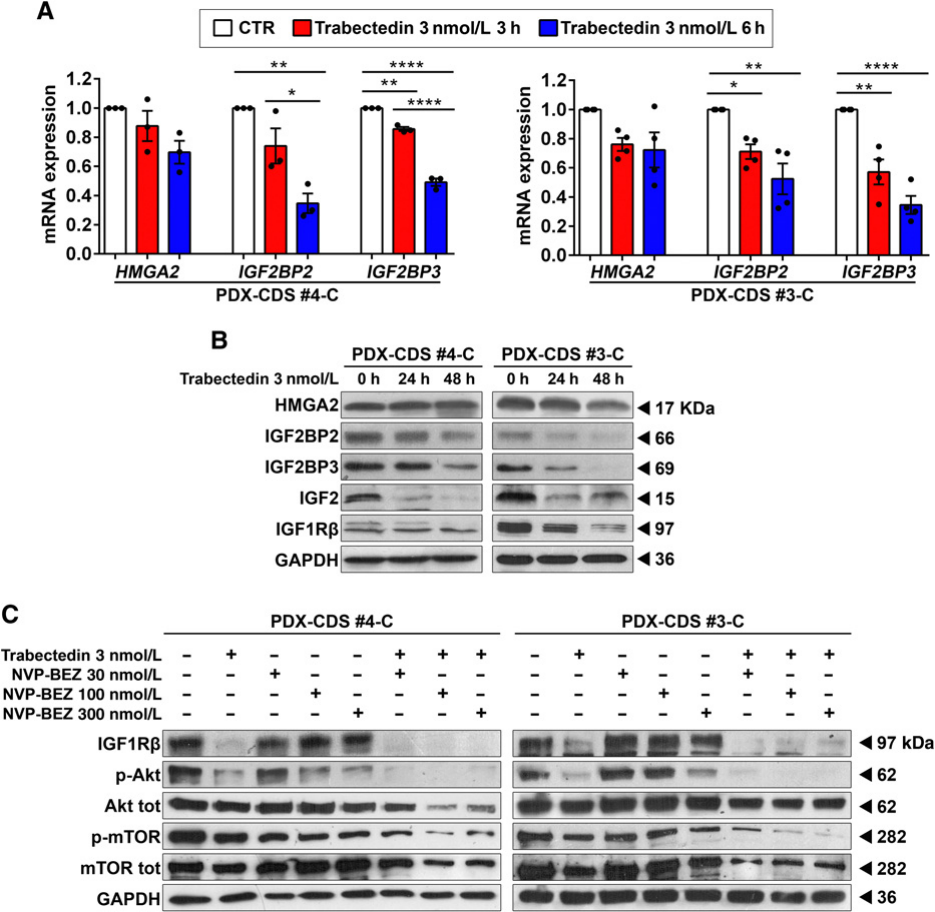
Trabectedin impairs the HMGA2/IGF2BP2–3/IGF network and inhibits AKT pathway activity in combination with NVP-BEZ235 treatment. **A** and **B,** qRT-PCR analysis of *HMGA2/IGF2BP2–3* mRNA expression (**A**) and Western blot analysis of HMGA2/IGF2BP2–3/IGF2/IGF1R protein expression (**B**) in PDX-CDS#4-C and PDX-CDS#3-C cells treated with 3 nmol/L trabectedin for the indicated time. At least three independent biological replicates were performed. One representative immunoblot is shown. For qRT-PCR, the data represent the mean ± SE. *, *P* < 0.05; **, *P* < 0.01; ****, *P* < 0.0001, one-way ANOVA. Each dot represents an independent experiment. *GAPDH* was used as a reference gene. **C,** Western blot analysis showing activation of IGF1R and the downstream mediator AKT in PDX-CDS#4-C and PDX-CDS#3-C cells treated with 3 nmol/L trabectedin for 24 hours with or without a subsequent 24 hours treatment with different doses of NVP-BEZ235. Three independent biological replicates were performed. One representative immunoblot is shown. GAPDH was employed for normalization purposes.

**Figure 5.**
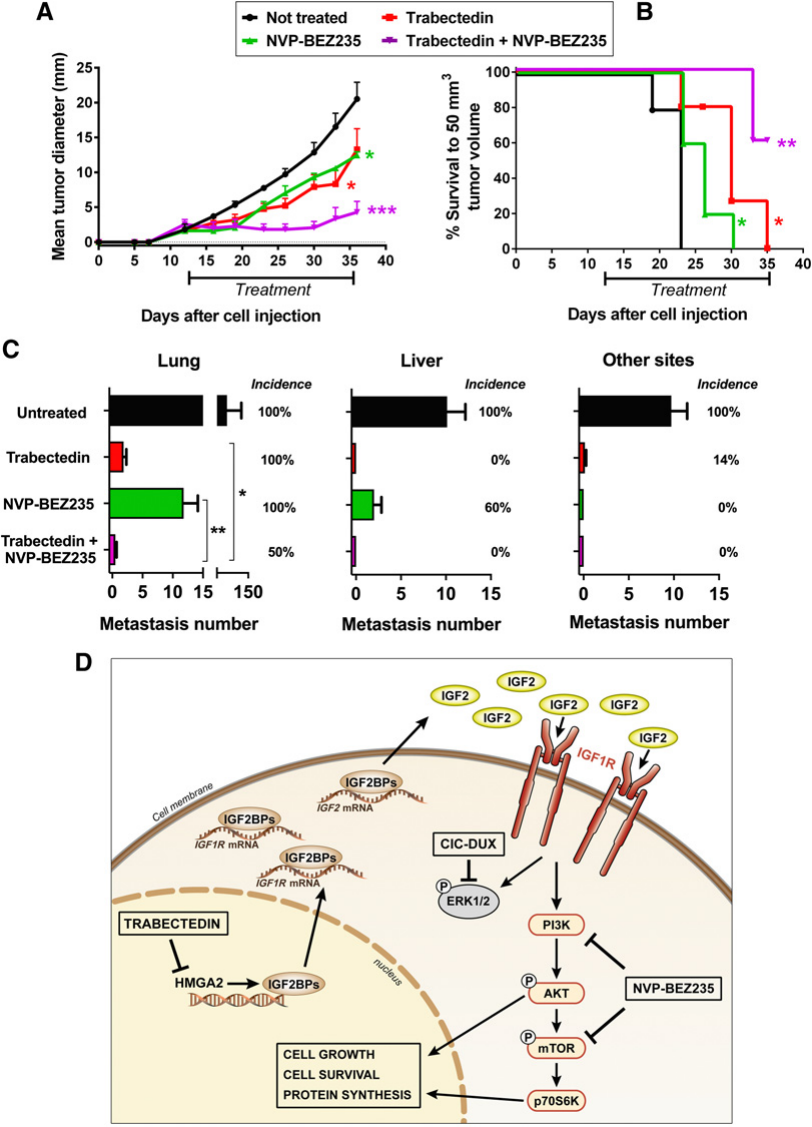
Efficacy of combined treatment with trabectedin and NVP-BEZ235 against CDS tumor growth and metastasis and schematic representation of the CDS-specific HMGA2/IGF2BPs/IGF2/IGF1R/AKT-mTOR pathway. **A,** Inhibition of PDX-CDS #4-C tumor growth after treatments with trabectedin and/or NVP-BEZ235. Significant reduction in tumor growth was observed after single treatments (*, *P* < 0.05, Student *t* test) but the inhibition increased after combination of the two drugs (significance was at least ***, *P* < 0.001, Student *t* test starting from day 23) or to single treatments (*, *P* < 0.05). Points, tumor diameter means (calculated as geometric mean in mm); bars, SE. Drugs were administered as indicated in the Supplementary Material and Methods. **B,** Tumor-free survival curves of mice treated with trabectedin and/or NVP-BEZ235. Kaplan–Meier curves and Mantel–Cox tests (compared with untreated mice) are shown: *, *P* < 0.05; **, *P* < 0.01. **C,** Inhibition of PDX-CDS#4-C experimental metastases to the lungs, liver, and other sites (mainly interscapular adipose tissue and lymph nodes) after treatments with trabectedin and/or NVP-BEZ235, starting from 7 days after intravenous cell injection. All of the treated groups developed a significantly lower number of metastases than untreated mice by the nonparametric Mann–Whitney test (*P* < 0.01; *n* = 7 for untreated control group and trabectedin; *n* = 5 for NVP-BEZ235; *n* = 6 for the combination Trabectedin + NVP-BEZ235). In the lung, the combined treatment led to a significant lower number of metastases compared with trabectedin (*, *P* < 0.05), or with NVP-BEZ235 (**, *P* < 0.01) as single agents. Bars, metastasis number means and SE. Incidence of mice with metastasis to the different sites (mice with metastasis/total number of mice per group) is reported as percentage. **D,** In the nucleus, high expression of HMGA2 favors the transcription of *IGF2BP2* and *IGF2BP3*. In the cytoplasm, IGF2BP2 and IGF2BP3 directly bind to and stabilize *IGF2* and *IGF1R* mRNAs, which subsequently activate IGF1R signaling. The repression of MAPK signaling by the CIC-DUX4 fusion protein renders CDSs mainly dependent on the AKT/mTOR pathway. Trabectedin can impair HMGA2 activity by preventing its binding to promoters, thus inhibiting the transcription of its targets *IGF2BP2* and *IGF2BP3* and decreasing IGF2/IGF1R signaling. NVP-BEZ235 is a dual PI3K/mTOR inhibitor used in phase II clinical trials. The combination of trabectedin with NVP-BEZ235 synergistically inhibits tumor growth.

To further enhance treatment efficacy, we evaluated combinatorial therapies with conventional chemotherapeutic drugs that are used for the treatment of Ewing sarcoma and CDS (Supplementary Table S9). As expected, compared with Ewing sarcoma cells, CDS cells were substantially more chemoresistant to doxorubicin, irinotecan and etoposide but similarly sensitive to vincristine, ifosfamide, and trabectedin. Trabectedin activity was observed at lower concentrations compared with the other agents and at dosages that are easily achievable in patients. In addition, trabectedin was reported to be more effective in cells expressing HMGAs (18). We thus investigated the impact of trabectedin on the expression of HMGA2 and its targets in CDS cells. Short-term exposure of PDX-CDS#4-C and PDX-CDS#3-C cells to trabectedin caused a dose-dependent decrease in the mRNA expression of the HMGA2 targets *IGF2BP2/IGF2BP3* (Fig. 4A) but did not significantly affect the mRNA expression of *HMGA2* itself, in keeping with the fact that trabectedin reduces the binding of HMGAs to the promoters of their target genes rather than altering its expression (18). Consistently, 24- to 48-hour exposure of PDX-CDS#4-C and PDX-CDS#3-C cells to trabectedin repressed IGF2BP2/3 protein expression (Fig. 4B) and, as a consequence, IGF2 and IGF1Rβ (42). This evidence strengthens the rationale for testing trabectedin in combination with inhibitors of the PI3K/mTOR pathway. As a proof of principle, we used the dual inhibitor NVP-BEZ235. Activation of IGF1R/AKT/mTOR signaling was dramatically inhibited upon combined treatment with trabectedin and NVP-BEZ235 (Fig. 4C). As a single agent, trabectedin efficiently inhibited pAKT but only partially affected the phosphorylation of mTOR (Fig. 4C). This result is in line with the fact that mTOR receives inputs from multiple signaling pathways in addition to AKT signaling (43) and it further supports the need to combine trabectedin with dual PI3K/mTOR inhibitors to obtain complete abrogation of the entire AKT/mTOR pathway. Notably, when trabectedin is combined with NVP-BEZ235, we obtained synergistic effects *in vitro* (Supplementary Fig. S12) and remarkable abrogation of *in vivo* tumor growth (Fig. 5A). Compared with the untreated group, mice receiving the combined therapy displayed 80% inhibition of tumor growth by the end of the treatment regimen. Compared with single-agent treatments, the combination regimen resulted in 68% inhibition of tumor growth versus trabectedin and 66% inhibition versus NVP-BEZ235. Moreover, at the end of the experiment, the Kaplan–Meier survival curve for the combined treatment group was significantly different from that of both the untreated group and the groups receiving either monotherapy, with 60% of mice in the combined treatment group developing no tumors compared with none of the untreated mice or monotherapy-treated mice (Fig. 5B). IHC evaluation of AKT signaling confirmed the abrogation of the AKT/mTOR pathway in tumors treated with combined treatments (Supplementary Fig. S13). In addition, considering that CDSs are highly aggressive sarcomas and that Akt/mTOR signaling enhances cancer metastasis, we verified whether combined treatment was effective against CDS metastatic dissemination. After intravenous injection, PDX-CDS#4-C cells displayed multiorgan dissemination, involving the lung, liver, interscapular brown adipose tissue and lymph nodes in 100% of untreated mice (Fig. 5C). Treatment with trabectedin or NVP-BEZ235 as single agents produced a strong and widespread reduction in the metastatic burden. However, these agents were unable to reduce the incidence of lung metastasis (Fig. 5C). In contrast, when the two drugs were combined, liver and other-site metastasis were completely abolished, while lung metastasis was significantly reduced in both number and incidence compared with trabectedin or NVP-BEZ235 as single agents (*P* < 0.05 and *P* < 0.01, respectively, Mann–Whitney test; Fig. 5C).

## Discussion

Cancer therapy is now shifting from the broad and indiscriminate use of conventional cytotoxic drugs to a more patient-tailored therapeutic approach that considers the specific molecular and cellular features of individual tumors. In many types of cancer, genetic analyses allow for the identification of specific oncogenic drivers that may serve as novel therapeutic targets. Unfortunately, many sarcomas, including CDS, are driven by undruggable molecular alterations and have a low mutational burden, which has hampered therapeutic advancements in recent years. Thus, the field is driven by the identification of alternative experimental options that aid cancer drug treatment decisions. Establishment of preclinical models representing individual tumors is a crucial step in the development of more effective therapeutic strategies. In particular, PDX models constitute an important tool for the expansion of patient-derived specimens and boast advantages including closely resembling the original tumor samples at the morphologic and molecular levels (22) and maintaining heterogeneity in individual drug responses. However, for screening potential drugs, *in vitro* cultures of cancer cells are more suitable for the timely prioritization of actionable drug targets. In this study, we show how CDS-derived PDXs and PDX-derived cell lines maintain the most representative genetic and phenotypic features of the original tumors and highlight the feasibility of these models to determine pharmacologic vulnerabilities with a high-fidelity prediction of the *in vivo* response. Our work integrates the few CDS experimental models that are already available (35, 44, 45) with the development of cell lines that are generated from patient tumors rather than artificial genetic modifications. Comprehensive molecular characterization of PDXs and PDX-derived cell lines allows for the detection of relevant pathogenetic pathways in a reasonable time frame. Specifically, we first identified an HMGA2/IGF2BP2/IGF2BP3/IGF2 axis that sustained constitutive IGF1R signaling in CDS cells. A role for the IGF system in CDS has already been reported (44). Expression of the *CIC-DUX4* fusion gene in Kitra-SRS cells was associated with autocrine activation of the IGF1/IGF1R pathway and treatment with the IGF1R inhibitor linsitinib attenuated cell growth and IGF1-induced activation of IGF1R/AKT signaling *in vitro* and *in vivo*. Our data provide a step forward in the identification of the molecular mechanisms that drive the unusually high constitutive activation of the IGF system in CDS by supporting the roles of HMGA2 and IGF2BP2/IGF2BP3 in IGF1R signaling. HMGA2 expression was found to be higher in stem cells, where it regulates self-renewal, impairs differentiation, and independently predicts poor clinical outcomes in several tumors by targeting key oncogenic pathways (46), including IGF2BP2 and 3. These RNA-binding proteins, in turn, favor the expression of IGF2/IGF1R (17, 38), leading to distinctive AKT/mTOR activation. In fact, the ability of the CIC-DUX4 oncoprotein to repress MAPK pathway mediators (10) renders CDS mainly or completely dependent on AKT/mTOR signaling for growth. Lin and colleagues (10) recently proposed pharmacologic MAPK activation to therapeutically degrade the CIC-DUX4 fusion protein; here, we propose that the dual inhibition of AKT/mTOR signaling in combination with trabectedin is optimal. AKT/mTOR inhibitors are currently in phase I/II clinical trials for adult malignancies (47, 48) and drugs such as everolimus, alpelisib, and MK-2206 have also been tested against bone sarcomas with variable results. Their efficacy as single agents against CDS cells are limited *in vitro*, with the exception of the PI3K/mTOR inhibitor NVP-BEZ235 (49, 50), but combined treatment with two selective inhibitors of the AKT or mTOR pathway, such as alpelisib and everolimus, results in increased efficacy, supporting the need for dual inhibition. mTOR pathway plays a central role in regulating cancer progression and is controlled by multiple mechanisms in addition to AKT signaling (43). An integrated understanding of the relative importance of these signals is beyond the purpose of this article; however, our *in vitro* and *in vivo* evidence clearly indicates that in the context of CDS the simultaneous inhibition of both Akt and mTOR provides a therapeutic advantage. As a proof of principle, we used NVP-BEZ235, a drug that has been orally administered to patients with advanced solid tumors in phase I/II clinical studies (51–53), to demonstrate the sensitivity of CDS cell lines to combined treatment with AKT/mTOR inhibitors and trabectedin (54). The synergistic *in vitro* effect of this combination accurately predicts *in vivo* responses, further supporting the use of PDX-derived cell lines as good experimental models for drug screening. The rationale for testing trabectedin was based on the following: (i) trabectedin was the only drug among several tested that induced marked growth suppression against an *ex vivo* CIC-DUX4–expressing mouse model (9); (ii) it was approved for clinical use in patients with other soft tissue sarcomas that have proven to be resistant to standard chemotherapy and other targeted therapies and/or metastasis; and (iii) it has ability to impair the function of HMGA proteins (18), which may confer additional value in the context of CDS. HMGA1 and 2 proteins are reported to bind the minor groove of DNA, alter chromatin structure and thus regulate the transcription of several genes by enhancing or suppressing the activity of transcription factors (55, 56). *HMGA2* was found to be aberrantly upregulated in the CDS in our dataset, as described previously (34, 57). In murine models of CDS obtained by transducing mouse embryonic mesenchymal cells with the *CIC-DUX4* gene (9), increased expression of *HMGA2* transcripts was also observed, while silencing of *CIC-DUX4* in our human CDS cells led to reduced expression of *HMGA2* and *IGF2BP* mRNAs. Whether HMGA2 is a direct target of the *CIC-DUX4* fusion product remains unknown, but these results collectively indicate that this molecule may play a critical role in CDS malignancy. Trabectedin was shown to displace HMGA proteins from HMGA-responsive promoters, impairing their transcriptional activity (18). Here, we confirmed that treatment with trabectedin did not modify HMGA2 protein expression levels but did affect the expression of the HMGA2 targets *IGF2BP2* and *IGF2BP3* (17, 58), resulting in modulated expression and activation of the IGF2/IGF1R signaling pathway (17, 42). Combined treatment with trabectedin and the AKT/mTOR inhibitor NVP-BEZ235 abolished the activity of the HMGA2/IGF2BPs/IGF2/IGF1R/AKT axis and showed powerful antitumor efficacy against CDS PDX-derived cell lines *in vitro* and *in vivo*. In particular, the drug combination was effective either against tumor growth or against metastasis, the major life-threatening clinical problem.

Overall, we uncovered an HMGA2/IGF2BPs/IGF2/IGF1R/AKT-mTOR functional pathway that characterizes CDS and renders the tumor particularly sensitive to combined treatment with trabectedin and AKT/mTOR inhibitors (schematically represented in Fig. 5D). The development of representative experimental models (PDXs and PDX-derived cell lines) even endowed with experimental multiorgan metastatic ability, has helped in revealing a mechanism-based therapeutic strategy to fight this lethal cancer.

## Authors' Disclosures

M. Carrabotta reports grants from AIRC, Horizon 2020-IMI2-ITCC-P4, and ERANET TRANSCAN-2_TORPEDO during the conduct of the study; non-financial support from PharmaMar and grants from Italian Ministry of Health outside the submitted work. M.A. Laginestra reports grants from AIRC, Horizon2020-IMI2-ITCC-P4, and ERANET TRANSCAN-2_TORPEDO during the conduct of the study; non-financial support from PharmaMar and grants from HORIZON 2020 SELNET outside the submitted work. G. Durante reports grants from AIRC, Horizon2020-IMI2-ITCC-P4, and ERANET TRANSCAN-2_TORPEDO during the conduct of the study; non-financial support from PharmaMar outside the submitted work. C. Mancarella reports grants from AIRC, Horizon2020-IMI2-ITCC-P4, and ERANET TRANSCAN-2_TORPEDO during the conduct of the study; non-financial support from PharmaMar outside the submitted work. L. Landuzzi reports grants from AIRC, Horizon2020-IMI2-ITCC-P4, and ERANET TRANSCAN-2_TORPEDO during the conduct of the study; non-financial support from PharmaMar outside the submitted work. A. Parra reports grants from AIRC, Horizon2020-IMI2-ITCC-P4, and ERANET TRANSCAN-2_TORPEDO during the conduct of the study; non-financial support from PharmaMar outside the submitted work. L. Toracchio reports grants from AIRC, Horizon2020-IMI2-ITCC-P4, and ERANET TRANSCAN-2_TORPEDO during the conduct of the study; non-financial support from PharmaMar outside the submitted work. A. De Feo reports grants from AIRC, Horizon2020-IMI2-ITCC-P4, and ERANET TRANSCAN-2_TORPEDO during the conduct of the study; non-financial support from PharmaMar and grants from Italian Ministry of Health outside the submitted work. V. Giusti reports grants from AIRC, Horizon2020-IMI2-ITCC-P4, and ERANET TRANSCAN-2_TORPEDO during the conduct of the study; non-financial support from PharmaMar and grants from Italian Ministry of Health outside the submitted work. M. Pasello reports grants from AIRC, HORIZON2020-IMI2-ITCC-P4, and ERANET TRANSCAN-2_TORPEDO during the conduct of the study; non-financial support from PharmaMar outside the submitted work. A. Righi reports grants from AIRC, Horizon2020-IMI2-ITCC-P4, and ERANET TRANSCAN-2_TORPEDO during the conduct of the study; non-financial support from PharmaMar outside the submitted work. E. Palmerini reports grants from AIRC, Horizon2020-IMI2-ITCC-P4, and ERANET-TRANSCAN2-TORPEDO during the conduct of the study; non-financial support and other support from PharmaMar; non-financial support from Pfizer, Bristol Myers Squibb; personal fees from Daiichi Sankyo, SynOx Therapeutics, Deciphera, and Eusa Pharma outside the submitted work. D.M. Donati reports grants from AIRC, Horizon2020-IMI2-ITCC-P4, and ERANET TRANSCAN-2_TORPEDO during the conduct of the study; non-financial support from PharmaMar and personal fees from Zimmer Biomet outside the submitted work. M.C. Manara reports grants from AIRC, Horizon 2020-IMI2-ITCC-P4, and ERANET TRANSCAN-2_TORPEDO during the conduct of the study; non-financial support from PharmaMar outside the submitted work. K. Scotlandi reports grants from Italian Association for Cancer Research (AIRC), Horizon2020-IMI2-ITCC-P4, and Eranet-Transcan2-TORPEDO during the conduct of the study; non-financial support from Pharmamar outside the submitted work. No disclosures were reported by the other authors.

## Supplementary Material

Supplementary Data

Supplementary Data
