# Supplementary material for: Integrated Molecular Characterization of Patient-Derived Models Reveals Therapeutic Strategies for Treating CIC-DUX4 Sarcoma
Source: Cancer Res. 2021 Dec 13;82(4):708–20. doi: 10.1158/0008-5472.CAN-21-1222 (PMC9359717; doi:10.1158/0008-5472.CAN-21-1222)
Supplement: Supplementary Data [file can-21-1222_supplementary_data_supp1-9.pdf]

Supplementary Tables S1-S9

Integrated molecular characterization of patient-derived models reveals therapeutic strategies for treating CIC-DUX4 sarcoma

Marianna Carrabotta, Maria Antonella Laginestra, Giorgio Durante, Caterina Mancarella, Lorena Landuzzi, Alessandro Parra, Francesca Ruzzi, Lisa Toracchio, Alessandra De Feo, Veronica Giusti, Michela Pasello, Alberto Righi, Pier-Luigi Lollini, Emanuela Palmerini, Davide Maria Donati, Maria Cristina Manara* and Katia Scotlandi*.

*Corresponding authors. Email: mariacristina.manara@ior.it; katia.scotlandi@ior.it

Supplementary Table S1. 537 differentially expressed genes between CDS and EWS.

Genes upregulated in CDS sorted according to fold change			
**Gene_Symbol**	**log_2FoldChange**	**FDR**	**Gene_Description**
ETV4	11.760	1.3965E-123	ETS variant transcription factor 4
UNC13A	7.594	4.17666E-38	unc-13 homolog A
BAI1	7.015	9.09419E-27	Adhesion G protein-coupled receptor B1
SHC4	6.717	1.84553E-32	SHC adaptor protein 4
ARVCF	6.595	1.07311E-38	ARVCF delta catenin family member
SHC3	6.548	7.15264E-37	SHC adaptor protein 3
NPTX1	6.365	7.24115E-18	neuronal pentraxin 1
DUSP4	6.143	2.16161E-21	dual specificity phosphatase 4
COL9A3	5.735	6.61345E-12	collagen type IX alpha 3 chain
SPON2	5.728	8.89075E-25	spondin 2
NHSL1	5.698	8.32907E-15	NHS like 1
FOXL1	5.653	1.27168E-16	forkhead box L1
WSCD1	5.603	3.42545E-18	WSC domain containing 1
FBN2	5.579	1.89306E-14	fibrillin 2
ACSF2	5.548	1.73298E-21	acyl-CoA synthetase family member 2
CBS	5.519	2.00629E-22	cystathionine beta-synthase
IGF2	5.456	3.04612E-11	insulin like growth factor 2
LAMC3	5.403	3.51557E-15	laminin subunit gamma 3
GCNT2	5.395	9.57474E-22	glucosaminyl (N-acetyl) transferase 2 (I blood group)
ZNF423	5.284	2.59411E-25	zinc finger protein 423
FOSL1	5.280	4.05737E-30	FOS like 1, AP-1 transcription factor subunit
FLT4	5.279	3.17374E-25	fms related tyrosine kinase 4
SPRED3	5.222	5.23642E-25	sprouty related EVH1 domain containing 3
POLE	5.178	4.91011E-25	DNA polymerase epsilon, catalytic subunit
HIF3A	5.167	1.00239E-19	hypoxia inducible factor 3 subunit alpha
ETV1	5.023	1.06894E-24	ETS variant transcription factor 1

BCL11A	5.012	3.39121E-13	BAF chromatin remodeling complex subunit BCL11A
AHRR	4.992	4.67408E-08	aryl-hydrocarbon receptor repressor
SHROOM3	4.788	1.70664E-16	shroom family member 3
GPR37	4.769	4.59207E-17	G protein-coupled receptor 37
MAP2	4.768	4.79487E-11	microtubule associated protein 2
MAPK8IP3	4.679	1.30294E-10	mitogen-activated protein kinase 8 interacting protein 3
IGF2BP3	4.587	2.41737E-13	insulin like growth factor 2 mRNA binding protein 3
ICOSLG	4.583	6.3191E-12	inducible T cell costimulator ligand
PLCH1	4.580	2.59721E-14	phospholipase C eta 1
LRRTM4	4.476	2.58428E-06	leucine rich repeat transmembrane neuronal 4
MCC	4.421	2.0877E-09	MCC regulator of WNT signaling pathway
PXDN	4.358	5.54308E-21	peroxidasin
LIF	4.345	5.21365E-10	LIF interleukin 6 family cytokine
MAMLD1	4.344	1.4813E-17	mastermind like domain containing 1
PTPN5	4.299	1.28128E-07	protein tyrosine phosphatase non-receptor type 5
F2RL1	4.254	4.777E-09	F2R like trypsin receptor 1
THSD7B	4.250	5.57756E-07	thrombospondin type 1 domain containing 7B
MAST1	4.231	2.4505E-09	microtubule associated serine/threonine kinase 1
BACH2	4.189	4.99661E-08	BTB domain and CNC homolog 2
NPTX2	4.182	4.24605E-07	neuronal pentraxin 2
KRT18	4.181	4.32247E-09	keratin 18
TLL2	4.178	7.68654E-17	tolloid like 2
LGR4	4.154	6.73383E-11	leucine rich repeat containing G protein-coupled receptor 4
PTPRN2	4.054	1.39949E-06	protein tyrosine phosphatase receptor type N2
EBF2	4.049	6.38198E-07	EBF transcription factor 2
CHD5	4.047	2.48727E-06	chromodomain helicase DNA binding protein 5
CDH2	4.025	1.42424E-06	cadherin 2
RNF157	4.010	4.75197E-07	ring finger protein 157
PTX3	3.955	3.53096E-07	pentraxin 3
C22ORF34	3.930	2.4035E-05	chromosome 22 open reading frame 34

CAPN14	3.926	8.01391E-08	calpain 14
FGF18	3.873	2.53477E-06	fibroblast growth factor 18
CELSR3	3.852	1.00436E-09	cadherin EGF LAG seven-pass G-type receptor 3
SHISA2	3.778	1.41052E-07	shisa family member 2
MID1	3.771	1.02881E-20	midline 1
FGFBP3	3.746	2.75458E-16	fibroblast growth factor binding protein 3
ARHGEF28	3.726	1.0902E-07	Rho guanine nucleotide exchange factor 28
LRP8	3.710	1.27865E-09	LDL receptor related protein 8
EME2	3.698	9.97156E-07	essential meiotic structure-specific endonuclease subunit 2
CHD7	3.694	7.52603E-07	chromodomain helicase DNA binding protein 7
SEMA6A	3.691	3.12669E-09	semaphorin 6A
CREB3L1	3.670	1.87748E-06	cAMP responsive element binding protein 3 like 1
PXMP2	3.651	3.26573E-17	peroxisomal membrane protein 2
ZNF215	3.651	1.37366E-07	zinc finger protein 215
HUNK	3.638	1.22949E-11	hormonally up-regulated Neu-associated kinase
SLC7A1	3.633	9.35799E-23	solute carrier family 7 member 1
EHD3	3.625	4.23099E-07	EH domain containing 3
KCTD1	3.618	1.01283E-13	potassium channel tetramerization domain containing 1
PHLDA2	3.607	6.76187E-12	pleckstrin homology like domain family A member 2
DNMT3B	3.606	2.37102E-11	DNA methyltransferase 3 beta
GNB3	3.590	7.5324E-11	G protein subunit beta 3
SPRY4	3.587	6.70602E-15	sprouty RTK signaling antagonist 4
MFSD2A	3.563	1.64507E-07	major facilitator superfamily domain containing 2A
LPCAT1	3.546	3.45747E-14	lysophosphatidylcholine acyltransferase 1
FOXC1	3.546	2.6677E-05	forkhead box C1
RLTPR	3.536	5.01216E-05	Capping protein regulator and myosin 1 linker 2
C19ORF26	3.535	1.22777E-07	CACN subunit beta associated regulatory protein
CCNE1	3.522	3.36049E-07	cyclin E1
IGSF9B	3.515	0.000261968	immunoglobulin superfamily member 9B
ETS1	3.511	1.18538E-13	ETS proto-oncogene 1. transcription factor

PER2	3.503	6.82859E-14	period circadian regulator 2
OLFM2	3.492	1.2355E-05	olfactomedin 2
KIF26A	3.476	7.5324E-11	kinesin family member 26A
PHLDA1	3.462	3.2609E-13	pleckstrin homology like domain family A member 1
AMOT	3.460	3.86978E-06	angiomotin
CXXC4	3.459	1.47446E-06	CXXC finger protein 4
GATA3	3.450	4.65607E-05	GATA binding protein 3
DPF1	3.448	2.36047E-05	double PHD fingers 1
CYP2S1	3.446	9.2153E-06	cytochrome P450 family 2 subfamily S member 1
ADAMTS9	3.421	6.02481E-09	ADAM metallopeptidase with thrombospondin type 1 motif 9
ASTN1	3.419	1.49737E-09	astrotactin 1
WNK2	3.414	0.000282351	WNK lysine deficient protein kinase 2
EFR3B	3.392	3.19234E-07	EFR3 homolog B
MAFF	3.383	1.40462E-07	MAF bZIP transcription factor F
HMGA1	3.381	1.07669E-07	high mobility group AT-hook 1
HS6ST2	3.366	3.56087E-05	heparan sulfate 6-O-sulfotransferase 2
FSD1	3.364	0.000123216	fibronectin type III and SPRY domain containing 1
FASN	3.347	1.10023E-08	fatty acid synthase
TCF7L1	3.342	1.59353E-05	transcription factor 7 like 1
PDZD2	3.340	2.15432E-07	PDZ domain containing 2
FGD5	3.320	1.3058E-06	FYVE. RhoGEF and PH domain containing 5
EFCAB4A	3.319	9.40204E-06	Calcium release activated channel regulator 2B
PKNOX2	3.315	2.98854E-06	PBX/knotted 1 homeobox 2
ECHDC3	3.308	9.04687E-12	enoyl-CoA hydratase domain containing 3
LMTK3	3.294	0.000195249	lemur tyrosine kinase 3
NYAP1	3.287	7.85702E-09	neuronal tyrosine phosphorylated phosphoinositide-3-kinase adaptor 1
UBALD2	3.279	2.05951E-21	UBA like domain containing 2
HAGHL	3.269	9.76275E-05	hydroxyacylglutathione hydrolase like
WFDC1	3.256	7.77643E-05	WAP four-disulfide core domain 1
ZSWIM4	3.246	6.92098E-10	zinc finger SWIM-type containing 4

PAPSS2	3.241	5.49748E-05	3'-phosphoadenosine 5'-phosphosulfate synthase 2
RHBDL1	3.241	1.64562E-05	rhomboid like 1
DOCK5	3.240	8.39952E-18	dedicator of cytokinesis 5
ERBB3	3.223	0.000275587	erb-b2 receptor tyrosine kinase 3
GK	3.221	0.000140331	glycerol kinase
UBASH3B	3.213	1.96387E-07	ubiquitin associated and SH3 domain containing B
RPS6KL1	3.212	0.00013282	ribosomal protein S6 kinase like 1
WNK3	3.207	6.35598E-08	WNK lysine deficient protein kinase 3
RGS9	3.206	5.70864E-05	regulator of G protein signaling 9
EPHA2	3.182	4.87101E-10	EPH receptor A2
LIPG	3.178	0.001265498	lipase G. endothelial type
RPS6KA2	3.175	5.83513E-05	ribosomal protein S6 kinase A2
PRSS30P	3.174	0.000158946	serine protease 30. pseudogene
RASIP1	3.172	2.72322E-07	Ras interacting protein 1
HAS3	3.166	1.29149E-08	hyaluronan synthase 3
TIAM1	3.164	1.66784E-05	TIAM Rac1 associated GEF 1
KCNK5	3.155	0.000150136	potassium two pore domain channel subfamily K member 5
TBX1	3.154	0.000609787	T-box transcription factor 1
EVA1A	3.154	0.000117276	eva-1 homolog A. regulator of programmed cell death
EP400	3.150	4.33258E-06	E1A binding protein p400
DZIP1	3.130	1.18095E-06	DAZ interacting zinc finger protein 1
ETV5	3.126	3.966E-08	ETS variant transcription factor 5
GPC6	3.122	9.76884E-29	glypican 6
SPRY2	3.115	6.0472E-05	sprouty RTK signaling antagonist 2
DNAH17	3.097	3.65079E-05	dynein axonemal heavy chain 17
EPHB3	3.097	1.13501E-05	EPH receptor B3
TFAP2A	3.094	0.00057346	transcription factor AP-2 alpha
SAPCD2	3.093	6.75819E-05	suppressor APC domain containing 2
ALMS1P	3.090	5.54743E-05	ALMS1P pseudogene 1
ADAM11	3.081	0.000124746	ADAM metallopeptidase domain 11

NETO2	3.071	1.91936E-05	neuropilin and tolloid like 2
ASB9	3.058	2.99708E-07	ankyrin repeat and SOCS box containing 9
CARD10	3.042	8.15124E-05	caspase recruitment domain family member 10
ZFPM1	3.040	2.11063E-06	zinc finger protein, FOG family member 1
SRCRB4D	3.015	0.000236492	Scavenger receptor cysteine rich family member with 4 domains
FGFR3	3.009	8.87361E-05	fibroblast growth factor receptor 3
RPH3AL	3.004	8.43034E-06	rabphilin 3A like (without C2 domains)
FAM64A	3.000	5.55893E-05	PICALM interacting mitotic regulator
HMGA2	2.995	0.002420399	high mobility group AT-hook 2
ARHGEF15	2.992	0.000180583	Rho guanine nucleotide exchange factor 15
IKBKE	2.988	8.48287E-15	inhibitor of nuclear factor kappa B kinase subunit epsilon
TIE1	2.988	3.96144E-06	tyrosine kinase with immunoglobulin like and EGF like domains 1
METRN	2.986	2.26325E-07	meteorin. glial cell differentiation regulator
TRABD2B	2.984	8.04612E-05	TraB domain containing 2B
SPIRE2	2.976	9.51925E-05	spire type actin nucleation factor 2
S1PR5	2.971	3.94199E-10	sphingosine-1-phosphate receptor 5
TERT	2.962	0.002961427	telomerase reverse transcriptase
MMP17	2.959	0.000476679	matrix metallopeptidase 17
GPRC5B	2.950	0.00031757	G protein-coupled receptor class C group 5 member B
CHDH	2.945	0.000350911	choline dehydrogenase
SPRED2	2.943	6.01071E-22	sprouty related EVH1 domain containing 2
CCSER1	2.919	0.000333563	coiled-coil serine rich protein 1
OSGIN1	2.913	0.000799821	oxidative stress induced growth inhibitor 1
NOVA2	2.900	0.000667651	NOVA alternative splicing regulator 2
HEY1	2.889	3.98487E-05	hes related family bHLH transcription factor with YRPW motif 1
CROCC	2.887	0.000140762	ciliary rootlet coiled-coil. rootletin
NES	2.886	2.25347E-05	nestin
PALM3	2.885	0.003135204	paralemmin 3
SH2B2	2.877	5.62637E-07	SH2B adaptor protein 2
MARCH3	2.863	3.92186E-06	membrane associated ring-CH-type finger 3

CHST7	2.860	8.76609E-06	carbohydrate sulfotransferase 7
NHS	2.831	0.000761758	NHS actin remodeling regulator
LAMA5	2.827	0.000646193	laminin subunit alpha 5
FRAS1	2.823	0.000313892	Fraser extracellular matrix complex subunit 1
MFI2	2.821	0.000567957	melanotransferrin
FAM57A	2.819	1.33173E-05	TLC domain containing 3A
FOXP4	2.817	1.91795E-07	forkhead box P4
FARP1	2.807	1.55204E-07	FERM. ARH/RhoGEF and pleckstrin domain protein 1
RHPN1	2.795	0.000351798	rhophilin Rho GTPase binding protein 1
EVI5L	2.795	5.3736E-08	ecotropic viral integration site 5 like
JPH3	2.793	0.003341913	junctophilin 3
ABCA3	2.784	5.73888E-05	ATP binding cassette subfamily A member 3
MAST2	2.774	3.79788E-07	microtubule associated serine/threonine kinase 2
YJEFN3	2.773	1.47854E-05	YjeF N-terminal domain containing 3
AKAP12	2.772	0.002042872	A-kinase anchoring protein 12
TBL1X	2.767	1.88206E-06	transducin beta like 1 X-linked
ARAP3	2.766	5.2788E-05	ArfGAP with RhoGAP domain. ankyrin repeat and PH domain 3
RBPMS2	2.766	0.000195249	RNA binding protein. mRNA processing factor 2
IGF2BP2	2.765	0.00025973	insulin like growth factor 2 mRNA binding protein 2
INF2	2.762	2.87397E-08	inverted formin. FH2 and WH2 domain containing
FANCA	2.762	1.1338E-06	FA complementation group A
CCDC154	2.761	0.001474173	coiled-coil domain containing 154
GRTP1	2.747	1.97204E-05	growth hormone regulated TBC protein 1
TRPS1	2.746	4.44523E-07	transcriptional repressor GATA binding 1
RHPN2	2.733	0.000826693	rhophilin Rho GTPase binding protein 2
ZNF732	2.729	0.000476679	zinc finger protein 732
FLJ42627	2.728	1.26307E-05	None
GOLGA7B	2.727	0.000768235	golgin A7 family member B
CCDC136	2.726	0.000698389	coiled-coil domain containing 136
ZNF219	2.719	0.000649993	zinc finger protein 219

FGFRL1	2.711	9.59284E-05	fibroblast growth factor receptor like 1
TENM4	2.709	0.004035128	teneurin transmembrane protein 4
KANK1	2.708	0.000681942	KN motif and ankyrin repeat domains 1
COL5A3	2.703	0.001192989	collagen type V alpha 3 chain
ITGA2B	2.700	0.002006315	integrin subunit alpha 2b
PCSK6	2.685	0.000351752	proprotein convertase subtilisin/kexin type 6
TRAPPC9	2.683	6.16187E-09	trafficking protein particle complex 9
RELN	2.682	0.006373037	reelin
PROSER2	2.679	6.65932E-09	proline and serine rich 2
PDE10A	2.676	0.000670514	phosphodiesterase 10A
NKD2	2.669	0.00334275	NKD inhibitor of WNT signaling pathway 2
FGF1	2.651	3.97479E-05	fibroblast growth factor 1
DGCR5	2.645	0.00789805	DiGeorge syndrome critical region gene 5
ADAMTSL4	2.640	0.003393836	ADAMTS like 4
DBNDD1	2.639	0.000527142	dysbindin domain containing 1
MMP28	2.637	0.001497782	matrix metallopeptidase 28
ABCC8	2.636	0.011798479	ATP binding cassette subfamily C member 8
TMEM145	2.635	0.003330801	transmembrane protein 145
ADAMTS16	2.635	0.00529827	ADAM metallopeptidase with thrombospondin type 1 motif 16
NAT8L	2.631	0.012998093	N-acetyltransferase 8 like
BDNF	2.627	0.000131699	brain derived neurotrophic factor
TGM1	2.626	0.000116211	transglutaminase 1
ARHGAP33	2.622	0.000627704	Rho GTPase activating protein 33
MPPED2	2.614	0.000299848	metallophosphoesterase domain containing 2
GLI3	2.611	0.000191378	GLI family zinc finger 3
HSPA12B	2.611	0.006134696	heat shock protein family A (Hsp70) member 12B
DYNC1I1	2.609	0.000952814	dynein cytoplasmic 1 intermediate chain 1
IL18R1	2.608	0.005050156	interleukin 18 receptor 1
GPT2	2.606	0.001352382	glutamic--pyruvic transaminase 2
HAPLN3	2.601	0.00243578	hyaluronan and proteoglycan link protein 3

TMEM132A	2.601	0.000301563	transmembrane protein 132A
FOXN3	2.596	1.58824E-10	forkhead box N3
RNF207	2.589	9.22688E-05	ring finger protein 207
MAP1S	2.583	3.11109E-08	microtubule associated protein 1S
PCGF2	2.581	0.000120342	polycomb group ring finger 2
RAI1	2.577	1.22716E-06	retinoic acid induced 1
TNIK	2.577	0.004996663	TRAF2 and NCK interacting kinase
FABP5	2.576	0.001420655	fatty acid binding protein 5
SCARF1	2.573	1.53257E-05	scavenger receptor class F member 1
UVSSA	2.564	9.36556E-05	UV stimulated scaffold protein A
SHCBP1	2.563	7.34218E-07	SHC binding and spindle associated 1
PDE8B	2.562	0.003764584	phosphodiesterase 8B
TRIOBP	2.562	5.74625E-05	TRIO and F-actin binding protein
NUAK1	2.561	0.000346942	NUAK family kinase 1
ACER2	2.560	8.51945E-05	alkaline ceramidase 2
ONECUT2	2.559	0.001522432	one cut homeobox 2
CDH26	2.558	0.000707949	cadherin 26
ADAMTS6	2.553	0.00146559	ADAM metallopeptidase with thrombospondin type 1 motif 6
ELMO3	2.544	0.000144622	engulfment and cell motility 3
CDT1	2.544	3.34238E-05	chromatin licensing and DNA replication factor 1
TMEM92	2.543	0.000238073	transmembrane protein 92
SLC4A11	2.542	7.97906E-05	solute carrier family 4 member 11
CRB2	2.542	0.003274574	crumbs cell polarity complex component 2
ADPRHL1	2.539	7.17966E-06	ADP-ribosylhydrolase like 1
KLHDC4	2.538	1.63499E-05	kelch domain containing 4
MAMDC4	2.533	2.48239E-05	MAM domain containing 4
PLXNB3	2.531	0.007076148	plexin B3
DUSP16	2.531	2.35921E-05	dual specificity phosphatase 16
LRRC3	2.527	1.14465E-05	leucine rich repeat containing 3
MST4	2.527	0.002731195	Serine/threonine kinase 26

BRICD5	2.525	0.001226943	BRICHOS domain containing 5
PCDHGA3	2.524	1.59563E-05	protocadherin gamma subfamily A. 3
ARSJ	2.520	0.003099943	arylsulfatase family member J
TNK2	2.515	0.000729477	tyrosine kinase non receptor 2
ULBP1	2.513	0.005391994	UL16 binding protein 1
SALL4	2.509	0.003303499	spalt like transcription factor 4
BAIAP2L1	2.508	0.001226583	BAR/IMD domain containing adaptor protein 2 like 1
ADAMTS3	2.507	0.010458867	ADAM metallopeptidase with thrombospondin type 1 motif 3
MAML3	2.501	6.47871E-06	mastermind like transcriptional coactivator 3
Genes downregulated in CDS sorted according to fold change			
**Gene_Symbol**	**log_2FoldChange**	**FDR**	**Gene_Description**
EPOR	-2.507	0.000420808	erythropoietin receptor
CYP27C1	-2.511	0.000411984	cytochrome P450 family 27 subfamily C member 1
FOS	-2.512	7.02467E-05	Fos proto-oncogene. AP-1 transcription factor subunit
RNASE6	-2.518	0.00349182	ribonuclease A family member k6
MARCH1	-2.520	0.000647918	membrane associated ring-CH-type finger 1
BTN3A3	-2.521	6.16009E-08	butyrophilin subfamily 3 member A3
PARP11	-2.523	4.8663E-07	poly(ADP-ribose) polymerase family member 11
PLP2	-2.524	0.001913292	proteolipid protein 2
ADCY2	-2.524	0.011411909	adenylate cyclase 2
FAM46A	-2.525	0.000799237	Terminal nucleotidyltransferase 5A
ZNF641	-2.528	8.18285E-13	zinc finger protein 641
SV2B	-2.530	0.001445494	synaptic vesicle glycoprotein 2B
ETS2	-2.531	0.000202895	ETS proto-oncogene 2. transcription factor
TLR7	-2.536	0.008345919	toll like receptor 7
MITF	-2.540	0.000177843	melanocyte inducing transcription factor
VAV1	-2.542	0.000512157	vav guanine nucleotide exchange factor 1
NSUN7	-2.547	0.000534626	NOP2/Sun RNA methyltransferase family member 7
SH3BGR	-2.561	0.000161563	SH3 domain binding glutamate rich protein

FERMT3	-2.563	3.82424E-08	fermitin family member 3
DLX3	-2.564	0.005701991	distal-less homeobox 3
DFNB59	-2.569	0.000470245	Pejvakin
CAMK2N1	-2.570	9.74134E-05	calcium/calmodulin dependent protein kinase II inhibitor 1
FRRS1	-2.571	0.004929788	ferric chelate reductase 1
TMEM37	-2.573	0.000322914	transmembrane protein 37
SNX18	-2.577	1.55363E-07	sorting nexin 18
FABP4	-2.579	0.001366036	fatty acid binding protein 4
SLCO3A1	-2.580	2.93709E-05	solute carrier organic anion transporter family member 3A1
ZIC2	-2.588	0.011766074	Zic family member 2
FZD1	-2.593	2.25354E-05	frizzled class receptor 1
GBP2	-2.606	0.002220295	guanylate binding protein 2
FCGRT	-2.609	1.70997E-05	Fc fragment of IgG receptor and transporter
TRAM1L1	-2.610	5.28332E-05	translocation associated membrane protein 1 like 1
ALPK1	-2.615	3.24051E-12	alpha kinase 1
SPG20	-2.619	2.954E-05	spartin
CLIC2	-2.619	5.80924E-05	chloride intracellular channel 2
TLR8	-2.625	0.00415351	toll like receptor 8
DRD1	-2.628	0.003909023	dopamine receptor D1
PPARG	-2.631	0.002006315	peroxisome proliferator activated receptor gamma
CASP1	-2.635	5.00701E-06	caspase 1
APOL6	-2.636	4.28762E-06	apolipoprotein L6
ZNRF2	-2.638	1.96592E-08	zinc and ring finger 2
CAPN3	-2.639	0.001156924	calpain 3
PDE5A	-2.643	0.000409519	phosphodiesterase 5A
SYN1	-2.648	0.001649744	synapsin I
DYRK3	-2.656	3.52584E-11	dual specificity tyrosine phosphorylation regulated kinase 3
RGS6	-2.657	0.003480601	regulator of G protein signaling 6
ME1	-2.662	0.000605209	malic enzyme 1
TRIM36	-2.663	0.005792153	tripartite motif containing 36

LMBRD1	-2.664	5.04669E-06	LMBR1 domain containing 1
C3AR1	-2.665	0.003908683	complement C3a receptor 1
DPY19L2P2	-2.666	0.000396696	DPY19L2 pseudogene 2
CLEC2B	-2.685	0.000314579	C-type lectin domain family 2 member B
MS4A6A	-2.690	0.004373059	membrane spanning 4-domains A6A
MAOA	-2.692	0.000322914	monoamine oxidase A
ZC3H6	-2.696	4.9198E-16	zinc finger CCCH-type containing 6
KIAA0825	-2.696	1.85624E-06	KIAA0825
SIAH3	-2.697	0.000636824	siah E3 ubiquitin protein ligase family member 3
CYBRD1	-2.703	0.001156924	cytochrome b reductase 1
LRRC49	-2.706	2.12928E-07	leucine rich repeat containing 49
GRIK2	-2.713	0.001996872	glutamate ionotropic receptor kainate type subunit 2
RASAL3	-2.716	0.000145232	RAS protein activator like 3
EVI2A	-2.727	0.000878756	ecotropic viral integration site 2A
DCHS2	-2.734	0.002031783	dachsous cadherin-related 2
KIF4B	-2.735	0.00196317	kinesin family member 4B
B2M	-2.749	5.80924E-05	beta-2-microglobulin
KIAA1107	-2.750	2.75501E-08	BTB domain containing 8
PLEKHA2	-2.754	1.42678E-05	pleckstrin homology domain containing A2
PQLC3	-2.762	1.59578E-07	None
PAX9	-2.771	0.003393836	paired box 9
RNU11	-2.773	5.3736E-08	RNA. U11 small nuclear
SECTM1	-2.775	0.000279539	secreted and transmembrane 1
EMP3	-2.779	5.26188E-08	epithelial membrane protein 3
NCAM2	-2.798	0.001329225	neural cell adhesion molecule 2
CRYL1	-2.804	1.95802E-06	crystallin lambda 1
LRRC10B	-2.809	0.001911353	leucine rich repeat containing 10B
GLRX	-2.809	0.000207518	glutaredoxin
IL2RG	-2.811	0.001689223	interleukin 2 receptor subunit gamma
UNC5A	-2.812	0.000702421	unc-5 netrin receptor A

NMI	-2.826	3.05235E-05	N-myc and STAT interactor
CTSS	-2.828	0.001165178	cathepsin S
FABP3	-2.828	0.00100322	fatty acid binding protein 3
MSRA	-2.832	0.000241366	methionine sulfoxide reductase A
TTC30B	-2.834	8.05499E-11	tetratricopeptide repeat domain 30B
CUBN	-2.839	2.45434E-08	cubilin
PRR5L	-2.841	0.000150504	proline rich 5 like
ADIRF	-2.841	0.002542963	adipogenesis regulatory factor
GRIP2	-2.846	7.46968E-05	glutamate receptor interacting protein 2
MBNL3	-2.858	5.07716E-07	muscleblind like splicing regulator 3
GPR34	-2.859	0.002666137	G protein-coupled receptor 34
C15ORF52	-2.865	1.23822E-06	Coiled-coil domain containing 9B
SLC24A2	-2.870	0.000261653	solute carrier family 24 member 2
BACE2	-2.879	0.00010002	beta-secretase 2
ANKRD42	-2.883	3.89537E-05	ankyrin repeat domain 42
TMEM205	-2.887	7.84494E-07	transmembrane protein 205
SLC38A2	-2.890	1.11439E-05	solute carrier family 38 member 2
NR3C2	-2.897	0.001319891	nuclear receptor subfamily 3 group C member 2
PRCP	-2.907	1.71022E-05	prolylcarboxypeptidase
AMPD3	-2.912	2.44218E-09	adenosine monophosphate deaminase 3
TTC30A	-2.931	6.73383E-11	tetratricopeptide repeat domain 30A
CDH8	-2.936	0.000598378	cadherin 8
PHYHD1	-2.940	3.79381E-07	phytanoyl-CoA dioxygenase domain containing 1
ZEB2	-2.942	5.33242E-06	zinc finger E-box binding homeobox 2
COX7A1	-2.943	0.000633718	cytochrome c oxidase subunit 7A1
SNORA68	-2.943	8.51826E-06	small nucleolar RNA. H/ACA box 68
EFS	-2.948	5.64932E-07	embryonal Fyn-associated substrate
CPVL	-2.953	0.000203959	carboxypeptidase vitellogenic like
CPE	-2.954	5.88159E-06	carboxypeptidase E
RHOH	-2.959	0.000646657	ras homolog family member H

FGL2	-2.960	8.59629E-05	fibrinogen like 2
BAI3	-2.967	3.62634E-06	Adhesion G protein-coupled receptor B3
IFI27L2	-2.975	2.03831E-06	interferon alpha inducible protein 27 like 2
ENPEP	-2.983	0.000104942	glutamyl aminopeptidase
NWD1	-2.989	0.002938443	NACHT and WD repeat domain containing 1
SYNE1	-2.989	6.9916E-08	spectrin repeat containing nuclear envelope protein 1
CD36	-2.992	0.000205073	CD36 molecule
TXNIP	-2.994	1.95001E-05	thioredoxin interacting protein
SLFN13	-2.997	6.08927E-08	schlafen family member 13
FLRT1	-3.002	0.000566622	fibronectin leucine rich transmembrane protein 1
FZD4	-3.005	2.66605E-06	frizzled class receptor 4
SMIM14	-3.012	3.21706E-07	small integral membrane protein 14
ABHD6	-3.023	3.6146E-06	abhydrolase domain containing 6
CACNB2	-3.033	0.000414544	calcium voltage-gated channel auxiliary subunit beta 2
RNASE1	-3.034	3.96321E-05	ribonuclease A family member 1. pancreatic
JAK1	-3.037	3.28979E-05	Janus kinase 1
HSPB2	-3.042	1.60737E-05	heat shock protein family B (small) member 2
GATM	-3.068	1.02398E-08	glycine amidinotransferase
SPR	-3.074	3.06587E-08	sepiapterin reductase
ADCY10P1	-3.076	5.95006E-05	ADCY10 pseudogene 1
SNORA12	-3.081	2.67856E-09	small nucleolar RNA. H/ACA box 12
DAPK1	-3.082	4.40947E-05	death associated protein kinase 1
ATP8B1	-3.086	3.38025E-07	ATPase phospholipid transporting 8B1
A2M	-3.091	0.000229103	alpha-2-macroglobulin
LITAF	-3.096	1.55599E-06	lipopolysaccharide induced TNF factor
LRRN2	-3.102	0.000215109	leucine rich repeat neuronal 2
FAM13C	-3.119	6.09889E-05	family with sequence similarity 13 member C
MAPT-IT1	-3.124	0.00054075	MAPT intronic transcript 1
TG	-3.134	0.000100961	thyroglobulin
AMY2B	-3.136	1.13433E-06	amylase alpha 2B

OAS1	-3.139	2.40333E-05	2'-5'-oligoadenylate synthetase 1
GDF6	-3.145	0.000510911	growth differentiation factor 6
SNORA63	-3.145	5.96534E-07	small nucleolar RNA. H/ACA box 63
FAM84B	-3.151	0.000134081	LRAT domain containing 1
EGF	-3.169	0.000638217	epidermal growth factor
C5	-3.184	1.61934E-09	complement C5
CDC37L1	-3.187	5.93999E-08	cell division cycle 37 like 1
CLMN	-3.204	4.61841E-09	calmin
GLCE	-3.220	4.53067E-08	glucuronic acid epimerase
MAPT	-3.247	9.18562E-05	microtubule associated protein tau
ATP1A1	-3.249	6.59387E-07	ATPase Na+/K+ transporting subunit alpha 1
ZDHHC21	-3.259	5.0615E-06	zinc finger DHHC-type containing 21
NEK11	-3.260	3.11667E-08	NIMA related kinase 11
LONRF1	-3.273	8.94766E-07	LON peptidase N-terminal domain and ring finger 1
DSEL	-3.275	4.3328E-09	dermatan sulfate epimerase like
CDKL2	-3.279	7.31675E-05	cyclin dependent kinase like 2
DUOXA1	-3.286	3.65312E-09	dual oxidase maturation factor 1
TOM1L1	-3.304	1.79416E-05	target of myb1 like 1 membrane trafficking protein
GAS1	-3.312	1.01577E-07	growth arrest specific 1
SYDE2	-3.341	5.12967E-06	synapse defective Rho GTPase homolog 2
KL	-3.347	8.84624E-05	klotho
GABARAPL1	-3.377	3.23881E-09	GABA type A receptor associated protein like 1
IL1RAP	-3.382	2.79639E-05	interleukin 1 receptor accessory protein
DPF3	-3.427	9.76988E-10	double PHD fingers 3
ALCAM	-3.437	1.23982E-07	activated leukocyte cell adhesion molecule
APLF	-3.440	7.20671E-17	aprataxin and PNKP like factor
TMEM178B	-3.490	8.06667E-07	transmembrane protein 178B
NLGN4X	-3.496	2.08432E-05	neuroligin 4 X-linked
SLC1A4	-3.496	1.13501E-05	solute carrier family 1 member 4
PAK3	-3.499	5.11826E-07	p21 (RAC1) activated kinase 3

CACNA2D1	-3.506	5.0615E-06	calcium voltage-gated channel auxiliary subunit alpha2delta 1
DAPK2	-3.520	2.47387E-10	death associated protein kinase 2
SMPDL3A	-3.521	1.59563E-05	sphingomyelin phosphodiesterase acid like 3A
CAV2	-3.551	2.61315E-05	caveolin 2
NTNG1	-3.557	5.2205E-05	netrin G1
ZMAT1	-3.560	8.85813E-14	zinc finger matrin-type 1
SLC1A1	-3.560	6.51789E-06	solute carrier family 1 member 1
TAGAP	-3.578	1.64455E-06	T cell activation RhoGTPase activating protein
CCDC171	-3.614	2.93789E-08	coiled-coil domain containing 171
HIST1H2BI	-3.629	1.28045E-06	None
ABCA5	-3.637	5.34418E-07	ATP binding cassette subfamily A member 5
CSPG5	-3.638	2.85887E-07	chondroitin sulfate proteoglycan 5
ARHGEF6	-3.640	9.57007E-08	Rac/Cdc42 guanine nucleotide exchange factor 6
ZNF704	-3.649	7.00409E-08	zinc finger protein 704
HOXD8	-3.665	8.82406E-09	homeobox D8
TUBA4A	-3.667	8.40239E-09	tubulin alpha 4a
ALDH1A1	-3.680	1.11395E-05	aldehyde dehydrogenase 1 family member A1
ARTN	-3.686	7.34218E-07	artemin
GIMAP2	-3.689	1.22934E-11	GTPase. IMAP family member 2
TIPARP	-3.694	2.19142E-09	TCDD inducible poly(ADP-ribose) polymerase
FRK	-3.702	7.87639E-06	fyn related Src family tyrosine kinase
AKAP7	-3.721	7.41136E-08	A-kinase anchoring protein 7
GCNT4	-3.737	9.54365E-05	glucosaminyl (N-acetyl) transferase 4
CHRDL1	-3.764	9.67621E-08	chordin like 1
RNF141	-3.784	8.15888E-12	ring finger protein 141
NCKAP1L	-3.814	1.08339E-09	NCK associated protein 1 like
RNF182	-3.815	5.12025E-05	ring finger protein 182
LY96	-3.817	7.63149E-15	lymphocyte antigen 96
ARPP21	-3.844	7.76935E-06	cAMP regulated phosphoprotein 21
CUX2	-3.860	3.85037E-06	cut like homeobox 2

LRRC6	-3.866	2.1553E-09	leucine rich repeat containing 6
AKR1C3	-3.897	2.86517E-21	aldo-keto reductase family 1 member C3
C7ORF41	-3.901	1.54915E-27	Maturin. neural progenitor differentiation regulator homolog
SPTLC3	-3.913	3.91371E-06	serine palmitoyltransferase long chain base subunit 3
STEAP3	-3.942	1.03774E-09	STEAP3 metalloreductase
TENM1	-3.958	1.23722E-07	teneurin transmembrane protein 1
HIST1H2BH	-3.959	3.95365E-07	H2B clustered histone 9
ALX4	-3.968	2.76817E-10	ALX homeobox 4
HOXD9	-4.060	1.70359E-06	homeobox D9
FLJ43663	-4.064	2.63964E-19	Long intergenic non-protein coding RNA. p53 induced transcript
MYPN	-4.076	1.6271E-06	myopalladin
TMEM71	-4.089	1.44011E-07	transmembrane protein 71
HMCN1	-4.093	3.3861E-06	hemicentin 1
EGR2	-4.100	7.14597E-09	early growth response 2
DUOX1	-4.100	1.87153E-13	dual oxidase 1
CLSTN2	-4.106	4.04777E-07	calsyntenin 2
FREM1	-4.126	6.08927E-08	FRAS1 related extracellular matrix 1
KMO	-4.150	1.08233E-07	kynurenine 3-monooxygenase
ABCA10	-4.154	3.28349E-07	ATP binding cassette subfamily A member 10
PTPN13	-4.156	1.56112E-09	protein tyrosine phosphatase non-receptor type 13
HLF	-4.182	4.67739E-06	HLF transcription factor. PAR bZIP family member
FBXO32	-4.189	9.56926E-09	F-box protein 32
KDSR	-4.217	2.84948E-21	3-ketodihydrosphingosine reductase
POU3F2	-4.234	3.83039E-08	POU class 3 homeobox 2
SOX6	-4.316	6.42064E-08	SRY-box transcription factor 6
SPATA6L	-4.346	8.76202E-12	spermatogenesis associated 6 like
SCN9A	-4.402	1.87972E-08	sodium voltage-gated channel alpha subunit 9
NBPF3	-4.507	3.77893E-18	NBPF member 3
EPHA4	-4.512	1.00239E-19	EPH receptor A4
FAM49A	-4.531	9.86288E-13	family with sequence similarity 49 member A

ACTN2	-4.534	1.83106E-08	actinin alpha 2
GPR64	-4.558	1.28977E-10	Adhesion G protein-coupled receptor G2
PPP1R1A	-4.610	3.29777E-08	protein phosphatase 1 regulatory inhibitor subunit 1A
DLG2	-4.628	1.4813E-17	discs large MAGUK scaffold protein 2
SLFN11	-4.636	2.82102E-16	schlafen family member 11
SLC17A7	-4.645	2.39785E-10	solute carrier family 17 member 7
ID2	-4.660	2.80989E-14	inhibitor of DNA binding 2
PAQR5	-4.767	1.50158E-10	progestin and adipoQ receptor family member 5
ENPP1	-4.772	6.05426E-16	ectonucleotide pyrophosphatase/phosphodiesterase 1
SLC24A3	-4.827	2.31423E-14	solute carrier family 24 member 3
TNFAIP6	-4.831	2.01233E-08	TNF alpha induced protein 6
G0S2	-4.879	1.97518E-11	G0/G1 switch 2
CDH23	-4.914	8.62996E-10	cadherin related 23
LDB2	-4.916	1.00436E-09	LIM domain binding 2
CPEB2	-4.940	4.09884E-23	cytoplasmic polyadenylation element binding protein 2
IKZF2	-5.008	6.23684E-64	IKAROS family zinc finger 2
DCDC2	-5.025	2.48153E-10	doublecortin domain containing 2
PARM1	-5.037	2.68904E-12	prostate androgen-regulated mucin-like protein 1
FAM65C	-5.054	8.99828E-11	RIPOR family member 3
STEAP2	-5.074	3.28879E-12	STEAP2 metalloreductase
SLCO5A1	-5.245	3.52103E-09	solute carrier organic anion transporter family member 5A1
PTGER3	-5.434	5.83276E-18	prostaglandin E receptor 3
WBSCR17	-5.447	9.00048E-19	Polypeptide N-acetylgalactosaminyltransferase 17
STEAP1	-5.470	7.2838E-11	STEAP family member 1
DNAJC12	-5.737	6.27024E-25	DnaJ heat shock protein family (Hsp40) member C12
PCDH11X	-5.827	2.59294E-15	protocadherin 11 X-linked
XG	-5.854	3.29308E-19	Xg glycoprotein (Xg blood group)
BCL11B	-5.941	3.00542E-25	BAF chromatin remodeling complex subunit BCL11B
PRKCB	-5.979	3.18805E-23	protein kinase C beta
CYP26B1	-6.297	6.13239E-44	cytochrome P450 family 26 subfamily B member 1

SYT1	-6.999	3.06891E-27	synaptotagmin 1
LOXHD1	-7.423	3.38129E-24	lipoxygenase homology domains 1
PKP1	-7.716	1.41142E-35	plakophilin 1
ITM2A	-8.394	4.87103E-37	integral membrane protein 2A

Supplementary Table S2: 19 top gene sets enriched in CDS selected according to NES and FDR value.

GeneSet	SIZE	ES	NES	FDR q-val	LEADING-EDGE
BENPORATH_ES_1	60	0.5108	2.8900	0.0000	tags=40%, list=10%, signal=44%
SWEET_KRAS_TARGETS_DN	16	0.7364	2.7442	0.0000	tags=56%, list=5%, signal=59%
KIM_WT1_TARGETS_UP	70	0.4544	2.6232	0.0001	tags=41%, list=14%, signal=47%
MEISSNER_BRAIN_HCP_WITH_H3K4ME3_AND_H3K27ME3	190	0.3675	2.6552	0.0001	tags=47%, list=20%, signal=56%
MIKKELSEN_NPC_HCP_WITH_H3K27ME3	30	0.5564	2.5495	0.0005	tags=60%, list=13%, signal=68%
GOBERT_OLIGODENDROCYTE_DIFFERENTIATION_UP	117	0.3800	2.4916	0.0006	tags=67%, list=35%, signal=100%
KOBAYASHI_EGFR_SIGNALING_24HR_DN	55	0.4376	2.4120	0.0011	tags=73%, list=35%, signal=110%
MEISSNER_NPC_HCP_WITH_H3K4ME2	95	0.3894	2.4388	0.0011	tags=40%, list=13%, signal=45%
CAIRO_HEPATOBLASTOMA_CLASSES_UP	107	0.3707	2.3452	0.0021	tags=89%, list=53%, signal=184%
MIKKELSEN_MEF_HCP_WITH_H3K27ME3	46	0.4388	2.3384	0.0021	tags=50%, list=13%, signal=57%
MIYAGAWA_TARGETS_OF_EWSR1_ETS_FUSIONS_DN	44	0.4618	2.3334	0.0022	tags=59%, list=23%, signal=76%
RIGGI_EWING_SARCOMA_PROGENITOR_DN	33	0.4820	2.3060	0.0028	tags=48%, list=17%, signal=57%

MANALO_HYPOXIA_DN	62	0.410 4	2.310 6	0.002 8	tags=82%, list=44%, signal=143 %
WHITEFORD_PEDIATRIC_CANCER_MARKERS	21	0.546 2	2.304 1	0.002 8	tags=86%, list=35%, signal=131 %
REACTOME_DEGRADATION_OF_THE_EXTRACELLULAR_MATRIX	20	0.562 7	2.295 5	0.002 9	tags=50%, list=9%, signal=55%
MARZEC_IL2_SIGNALING_UP	28	0.507 6	2.254 3	0.004 2	tags=54%, list=18%, signal=65%
REACTOME_EXTRACELLULAR_MATRIX_ORGANIZATION	43	0.440 1	2.242 5	0.004 6	tags=51%, list=20%, signal=63%
FISCHER_G2_M_CELL_CYCLE	33	0.462 1	2.212 0	0.006 2	tags=67%, list=30%, signal=94%
AMIT_EGF_RESPONSE_120_HELA	15	0.593 3	2.177 2	0.008 7	tags=73%, list=26%, signal=98%

Supplementary Table S3: 71 genes contributing the most to the enriched pathway resulting from leading edge analysis. Genes are sorted according to fold change

Gene_Symbol	log_2FoldChange	FDR	Gene_Description
ETV4	11.760401626518	1.3964546334222E-123	ETS variant transcription factor 4
DUSP4	6.1426665075057	2.1616057175985E-21	dual specificity phosphatase 4
FOXL1	5.6528043911619	1.2716769949131E-16	forkhead box L1
CBS	5.5194828571222	2.0062909033775E-22	cystathionine beta-synthase
IGF2	5.455511875298	3.0461157925321E-11	insulin like growth factor 2
FOSL1	5.2797972980626	4.057366162365E-30	FOS like 1, AP-1 transcription factor subunit
ETV1	5.0225610480489	1.0689353307083E-24	ETS variant transcription factor 1
BCL11A	5.011596570579	3.3912093333587E-13	BAF chromatin remodeling complex subunit BCL11A
GPR37	4.7687931300266	4.5920706003758E-17	G protein-coupled receptor 37
IGF2BP3	4.5874019414371	2.4173656778691E-13	insulin like growth factor 2 mRNA binding protein 3
LIF	4.3447709392999	5.21365070365E-10	LIF interleukin 6 family cytokine
F2RL1	4.2536370862909	4.7769952881474E-09	F2R like trypsin receptor 1
MAST1	4.2308832656056	2.4505007723414E-09	microtubule associated serine/threonine kinase 1
LGR4	4.1542014800511	6.7338255077669E-11	leucine rich repeat containing G protein-coupled receptor 4
CDH2	4.0246436916968	1.4242420806361E-06	cadherin 2
PTX3	3.9545525385483	3.5309587241093E-07	pentraxin 3
LRP8	3.7100802826123	1.2786453035354E-09	LDL receptor related protein 8
CHD7	3.6941377067479	7.5260336551385E-07	chromodomain helicase DNA binding protein 7
SEMA6A	3.6906944203047	3.126689750454E-09	semaphorin 6A
SLC7A1	3.6330823080323	9.35799299388E-23	solute carrier family 7 member 1
PHLDA2	3.6074772726691	6.7618650156906E-12	pleckstrin homology like domain family A member 2
DNMT3B	3.6062147672349	2.3710154876667E-11	DNA methyltransferase 3 beta
SPRY4	3.5874079009553	6.7060150488084E-15	sprouty RTK signaling antagonist 4
FOXC1	3.5455057301854	2.6676950242157E-05	forkhead box C1
ETS1	3.5105047797881	1.1853830302583E-13	ETS proto-oncogene 1, transcription factor
PHLDA1	3.4617455964898	3.260900278127E-13	pleckstrin homology like domain family A member 1

CYP2S1	3.446006101576	9.215298873303E-06	cytochrome P450 family 2 subfamily S member 1
MAFF	3.3834470401982	1.4046177590462E-07	MAF bZIP transcription factor F
HMGA1	3.380781186171	1.0766888010731E-07	high mobility group AT-hook 1
TCF7L1	3.3421551232414	1.5935253657382E-05	transcription factor 7 like 1
EPHA2	3.1819874394559	4.8710051144466E-10	EPH receptor A2
LIPG	3.1776687688548	0.001265497580974	lipase G, endothelial type
HAS3	3.1657810033238	1.2914870676564E-08	hyaluronan synthase 3
TIAM1	3.1636689096068	1.6678395525573E-05	TIAM Rac1 associated GEF 1
EVA1A	3.1538993975835	0.000117275583486	eva-1 homolog A, regulator of programmed cell death
ETV5	3.125770239332	3.9659984663712E-08	ETS variant transcription factor 5
SPRY2	3.1152267059458	6.0472019093591E-05	sprouty RTK signaling antagonist 2
EPHB3	3.0968319577961	1.1350099492351E-05	EPH receptor B3
CARD10	3.0424828409897	8.1512377529782E-05	caspase recruitment domain family member 10
HMGA2	2.9948080226488	0.002420399478174	high mobility group AT-hook 2
S1PR5	2.9707454164807	3.94199151037E-10	sphingosine-1-phosphate receptor 5
GPRC5B	2.949803617675	0.000317569823878	G protein-coupled receptor class C group 5 member B
SPRED2	2.9431833435242	6.0107090606461E-22	sprouty related EVH1 domain containing 2
SH2B2	2.8769096339918	5.6263679883167E-07	SH2B adaptor protein 2
CHST7	2.8604155344902	8.7660943033666E-06	carbohydrate sulfotransferase 7
JPH3	2.7934207124788	0.003341912983513	junctophilin 3
AKAP12	2.7721746285642	0.002042872463018	A-kinase anchoring protein 12
IGF2BP2	2.7653010247409	0.000259730105399	insulin like growth factor 2 mRNA binding protein 2
PDE10A	2.6761932146219	0.000670513580406	phosphodiesterase 10A
FGF1	2.6511866407544	3.9747856652789E-05	fibroblast growth factor 1
DBNDD1	2.6394970210888	0.000527142449748	dysbindin domain containing 1
FABP5	2.576139079582	0.0014206547964	fatty acid binding protein 5
CDT1	2.5438482142457	3.3423803942192E-05	chromatin licensing and DNA replication factor 1
SALL4	2.5090064390418	0.003303498950733	spalt like transcription factor 4
SLC12A5	2.4918955995623	0.003103244064875	solute carrier family 12 member 5
PITPNC1	2.476586536991	6.9245566733432E-06	phosphatidylinositol transfer protein cytoplasmic 1

CSDC2	2.439721538913	8.170391242466E-05	cold shock domain containing C2
ROBO1	2.4315141909716	2.6155848976886E-05	roundabout guidance receptor 1
TMC6	2.4266604040924	0.000217514939552	transmembrane channel like 6
MAP3K9	2.3896688424269	0.00097988931301	mitogen-activated protein kinase kinase kinase 9
AUTS2	2.3778658946241	0.000317569823878	activator of transcription and developmental regulator AUTS2
SYNGR3	2.3518290283199	0.008321499121418	synaptogyrin 3
CACNA2D2	2.3504695920109	0.001044013396348	calcium voltage-gated channel auxiliary subunit alpha2delta 2
CPLX1	2.2100888579735	0.027812673840191	complexin 1
RAP1GAP2	2.2032503271706	0.007478591792378	RAP1 GTPase activating protein 2
ATP1B1	2.1715323874164	0.01152503207931	ATPase Na+/K+ transporting subunit beta 1
CLDN7	2.1707654097721	0.002664228296829	claudin 7
MAP2K3	2.1698766246712	4.0937550436091E-11	mitogen-activated protein kinase kinase 3
JAG2	2.1670695257321	0.00621785078474	jagged canonical Notch ligand 2
SPATA2L	2.120343330105	1.1350099492351E-05	spermatogenesis associated 2 like
TNFRSF12A	2.09997207781	6.9652208497099E-06	TNF receptor superfamily member 12A

Supplementary table S4: PDX-MI of CDS and EWS PDX models

Module	Field	CDS PDX-Models			
Clinical/patient	Patient ID	CDS#1	CDS#2	CDS#3	CDS#4
	gender	female	female	male	female
	age	adult	adult	adult	pediatric
	diagnosis	CIC-DUX sarcoma	CIC-DUX sarcoma	CIC-DUX sarcoma	CIC-DUX sarcoma
	Consent to share	Available to academic centers	Available to academic centers	Available to academic centers	Available to academic centers
	Status	dead	AWD	dead	dead
Clinical/tumor	Tumor ID	CDS#1-TUM	CDS#2-TUM	CDS#3-TUM	CDS#4-TUM
	Primary tumor tissue of origin	Soft tissue	Soft-tissue	Soft tissue	Soft tissue
	Primary, recurrence, metastasis	Primary	Primary	metastasis	Primary
	Specimen tumor tissue	thigh	spine	Lymph node	gluteus
	Specific markers	ETV4; CD99	ETV4; CD99	ETV4; CD99	ETV4; CD99
	Pre/post chemo	Post	Post	Post	Post
Model creation	PDX-ID	PDX-CDS#1	PDX-CDS#2	PDX-CDS#3	PDX-CDS#4
	Mouse strain (and source)	NSG (Charles River)	NSG (Charles River)	NSG (Charles River)	NSG (Charles River)
	Strain immune system humanized	no	no	no	no
	Tumor preparation	Tumor solid	Tumor solid	Tumor solid	Tumor solid
	Injection type and site	Sc (trans-scapular fat)	Sc (trans-scapular fat)	Sc (trans-scapular fat)	Sc (trans-scapular fat)
Model quality assurance	Tumor characterization technology	Histology and IHC	Histology and IHC	Histology and IHC	Histology and IHC
	Tumor confirmed not to be of mouse origin	Yes	Yes	Yes	Yes
	Passage QA performed	Passage P2	Passage P2	Passage P2	Passage P2
	Engraftment rate (PDX/number of mice)	100% (10/10)	100% (3/3)	100% (8/8)	100% (8/8)
	Median Engraftment time (weeks of latency of passages ≥ P3)	2	4	3	2

Module	*Field*	*EWS PDX-Models*		
Clinical/patient	Patient ID	EWS#2	EWS#4	EWS#5
	gender	male	female	male
	age	adult	pediatric	adult
	diagnosis	Ewing sarcoma	Ewing sarcoma	Ewing sarcoma
	Consent to share	Available to academic centers	Available to academic centers	Available to academic centers
	Status	Dead	Dead	Alive
Clinical/tumor	Tumor ID	EWS#2-TUM	EWS#4-TUM	EWS#5-TUM
	Primary tumor tissue of origin	soft	bone	bone
	Primary, recurrence, metastasis	Relapse	Primary	Primary
	Specimen tumor tissue	shoulder	pelvis	femur
	Specific markers	CD99	CD99	CD99
	Pre/post chemo	Post	Post	Post
Model creation	PDX-ID	PDX-EWS#2	PDX-EWS#4	PDX-EWS#5
	Mouse strain (and source)	NSG (Charles River)	NSG (Charles River)	NSG (Charles River)
	Strain immune system humanized	no	no	no
	Tumor preparation	Tumor solid	Tumor solid	Tumor solid
	Injection type and site	Sc (trans-scapular fat)	Sc (trans-scapular fat)	Sc (trans-scapular fat)
Model quality assurance	Tumor characterization technology	Histology and IHC	Histology and IHC	Histology and IHC
	Tumor confirmed not to be of mouse origin	Yes	Yes	Yes
	Passage QA performed	Passage P2	Passage P2	Passage P2
	Engraftment rate (PDX/number of mice)	100% (12/12)	100% (8/8)	80% (4/5)
	Median Engraftment time (weeks of latency of passages ≥ P3)	3	4	4

Abbreviations: AWD, alive with disease; NSG, NOD scid gamma; Sc, subcutaneous; IHC, immunohistochemistry

Supplementary Table S5: Confidence Interval ($CI_{95\%}$) for correlation coefficient computed on 3179 gene signature.

Var1	Var2	Rho	$CI_{95\%}$ (lower-upper)
CDS#1	PDX-CDS#1	0.95	0.946-0.954
CDS#1	PDX-CDS#1-C	0.93	0.924-0.935
PDX-CDS#1	PDX-CDS#1-C	0.95	0.946-0.954
CDS#3	PDX-CDS#3	0.79	0.774-0.805
CDS#3	PDX-CDS#3-C	0.81	0.795-0.824
PDX-CDS#3	PDX-CDS#3-C	0.95	0.946-0.954
CDS#4	PDX-CDS#4	0.92	0.913-0.926
CDS#4	PDX-CDS#4-C	0.88	0.870-0.889
PDX-CDS#4	PDX-CDS#4-C	0.92	0.913-0.926
CDS#1	CDS#3	0.85	0.838-0.861
CDS#1	PDX-CDS#3	0.87	0.859-0.880
CDS#1	PDX-CDS#3-C	0.89	0.881-0.898
PDX-CDS#1	CDS#3	0.8	0.785-0.814
PDX-CDS#1	PDX-CDS#3	0.89	0.881-0.898
PDX-CDS#1	PDX-CDS#3-C	0.9	0.892-0.908
PDX-CDS#1-C	CDS#3	0.77	0.753-0.786
PDX-CDS#1-C	PDX-CDS#3	0.88	0.870-0.889
PDX-CDS#1-C	PDX-CDS#3-C	0.92	0.913-0.926
CDS#1	CDS#4	0.9	0.892-0.908
CDS#1	PDX-CDS#4	0.88	0.870-0.889
CDS#1	PDX-CDS#4-C	0.85	0.838-0.861
PDX-CDS#1	CDS#4	0.85	0.838-0.861
PDX-CDS#1	PDX-CDS#4	0.89	0.881-0.898
PDX-CDS#1	PDX-CDS#4-C	0.86	0.849-0.870
PDX-CDS#1-C	CDS#4	0.83	0.817-0.842
PDX-CDS#1-C	PDX-CDS#4	0.87	0.859-0.880
PDX-CDS#1-C	PDX-CDS#4-C	0.89	0.881-0.898
CDS#3	CDS#4	0.87	0.859-0.880
CDS#3	PDX-CDS#4	0.82	0.806-0.833
CDS#3	PDX-CDS#4-C	0.73	0.710-0.748
PDX-CDS#3	CDS#4	0.84	0.827-0.852
PDX-CDS#3	PDX-CDS#4	0.89	0.881-0.898
PDX-CDS#3	PDX-CDS#4-C	0.83	0.817-0.842
PDX-CDS#3-C	CDS#4	0.86	0.849-0.870
PDX-CDS#3-C	PDX-CDS#4	0.9	0.892-0.908
PDX-CDS#3-C	PDX-CDS#4-C	0.92	0.913-0.926

Supplementary Table S6: Confidence Interval ($CI_{95\%}$) for correlation coefficient computed on 71 gene signature.

Var1	Var2	Rho	$CI_{95\%}$ (lower-upper)
CDS#1	PDX-CDS#1	0.94	0.895-0.965
CDS#1	PDX-CDS#1-C	0.93	0.878-0.960
PDX-CDS#1	PDX-CDS#1-C	0.92	0.862-0.954
CDS#3	PDX-CDS#3	0.71	0.544-0.822
CDS#3	PDX-CDS#3-C	0.74	0.586-0.842
PDX-CDS#3	PDX-CDS#3-C	0.92	0.862-0.954
CDS#4	PDX-CDS#4	0.95	0.912-0.971
CDS#4	PDX-CDS#4-C	0.90	0.829-0.942
PDX-CDS#4	PDX-CDS#4-C	0.89	0.813-0.936
CDS#1	CDS#3	0.75	0.600-0.848
CDS#1	PDX-CDS#3	0.84	0.734-0.905
CDS#1	PDX-CDS#3-C	0.87	0.781-0.924
PDX-CDS#1	CDS#3	0.68	0.503-0.802
PDX-CDS#1	PDX-CDS#3	0.83	0.718-0.899
PDX-CDS#1	PDX-CDS#3-C	0.83	0.718-0.899
PDX-CDS#1-C	CDS#3	0.64	0.450-0.774
PDX-CDS#1-C	PDX-CDS#3	0.81	0.688-0.887
PDX-CDS#1-C	PDX-CDS#3-C	0.84	0.734-0.905
CDS#1	CDS#4	0.77	0.629-0.861
CDS#1	PDX-CDS#4	0.74	0.586-0.842
CDS#1	PDX-CDS#4-C	0.81	0.688-0.887
PDX-CDS#1	CDS#4	0.77	0.629-0.861
PDX-CDS#1	PDX-CDS#4	0.78	0.644-0.868
PDX-CDS#1	PDX-CDS#4-C	0.81	0.688-0.887
PDX-CDS#1-C	CDS#4	0.73	0.572-0.835
PDX-CDS#1-C	PDX-CDS#4	0.71	0.544-0.822
PDX-CDS#1-C	PDX-CDS#4-C	0.83	0.718-0.899
CDS#3	CDS#4	0.72	0.558-0.829
CDS#3	PDX-CDS#4	0.64	0.450-0.774
CDS#3	PDX-CDS#4-C	0.63	0.437-0.767
PDX-CDS#3	CDS#4	0.76	0.615-0.855
PDX-CDS#3	PDX-CDS#4	0.81	0.688-0.887
PDX-CDS#3	PDX-CDS#4-C	0.85	0.749-0.912
PDX-CDS#3-C	CDS#4	0.78	0.644-0.868
PDX-CDS#3-C	PDX-CDS#4	0.79	0.658-0.874
PDX-CDS#3-C	PDX-CDS#4-C	0.87	0.781-0.924

Supplementary Table S7: Significance statistical test comparing intra-group correlation coefficients.

Var1	rho_Var1	Var2	rho_Var2	p-value	Bonferroni Correction
Paired comparisons based on 3179 gene signature					
CDS#1/PDX-CDS#1	0.95	CDS#1/PDX-CDS#1-C	0.93	0.2437	0.9999
CDS#1/PDX-CDS#1	0.95	PDX-CDS#1/PDX-CDS#1-C	0.95	1	1
CDS#1/PDX-CDS#1-C	0.93	PDX-CDS#1/PDX-CDS#1-C	0.95	0.2437	0.9999
CDS#3/PDX-CDS#3	0.79	CDS#3/PDX-CDS#3-C	0.81	0.2499	0.9999
CDS#3/PDX-CDS#3	0.79	PDX-CDS#3/PDX-CDS#3-C	0.95	1.23e-24	0.000
CDS#3/PDX-CDS#3-C	0.81	PDX-CDS#3/PDX-CDS#3-C	0.95	5.91e-20	0.000
CDS#4/PDX-CDS#4	0.92	CDS#4/PDX-CDS#4-C	0.88	0.01787	0.9999
CDS#4/PDX-CDS#4	0.92	PDX-CDS#4/PDX-CDS#4-C	0.92	1	1
CDS#4/PDX-CDS#4-C	0.88	PDX-CDS#4/PDX-CDS#4-C	0.92	0.01787	0.9999
Paired comparisons based on 71 gene signature					
**Var1**	**rho_Var1**	**Var2**	**rho_Var2**	**p-value**	**Bonferroni Correction**
CDS#1/PDX-CDS#1	0.94	CDS#1/PDX-CDS#1-C	0.93	0.9308	0.9999
CDS#1/PDX-CDS#1	0.94	PDX-CDS#1/PDX-CDS#1-C	0.92	0.8632	0.9999
CDS#1/PDX-CDS#1-C	0.93	PDX-CDS#1/PDX-CDS#1-C	0.92	0.9319	0.9999
CDS#3/PDX-CDS#3	0.71	CDS#3/PDX-CDS#3-C	0.74	0.8007	0.9999
CDS#3/PDX-CDS#3	0.71	PDX-CDS#3/PDX-CDS#3-C	0.92	0.0434	0.217
CDS#3/PDX-CDS#3-C	0.74	PDX-CDS#3/PDX-CDS#3-C	0.92	0.0741	0.371
CDS#4/PDX-CDS#4	0.95	CDS#4/PDX-CDS#4-C	0.90	0.6573	0.9999
CDS#4/PDX-CDS#4	0.95	PDX-CDS#4/PDX-CDS#4-C	0.89	0.5977	0.9999
CDS#4/PDX-CDS#4-C	0.90	PDX-CDS#4/PDX-CDS#4-C	0.89	0.9325	0.9999

Supplementary Table S8: Significance statistical test comparing inter-group correlation coefficients.

Var1	rho_Var1	Var2	rho_Var2	p-value	Bonferroni Correction
Paired comparisons based on 3179 gene signature					
CDS#1/PDX-CDS#1	0.95	CDS#3/PDX-CDS#3	0.79	1.156875e-201	1.041188e-200
CDS#1/PDX-CDS#1	0.95	CDS#3/PDX-CDS#3-C	0.81	1.53e-173	1.376541e-172
CDS#1/PDX-CDS#1	0.95	PDX-CDS#3/PDX-CDS#3-C	0.95	1	1
CDS#1/PDX-CDS#1-C	0.93	CDS#3/PDX-CDS#3	0.79	5.386639e-121	4.847975e-120
CDS#1/PDX-CDS#1-C	0.93	CDS#3/PDX-CDS#3-C	0.81	1.638163e-99	1.474347e-98
CDS#1/PDX-CDS#1-C	0.93	PDX-CDS#3/PDX-CDS#3-C	0.95	4.861093e-12	4.374984e-11
PDX-CDS#1/PDX-CDS#1-C	0.95	CDS#3/PDX-CDS#3	0.79	1.156875e-201	1.041188e-200
PDX-CDS#1/PDX-CDS#1-C	0.95	CDS#3/PDX-CDS#3-C	0.81	1.52949E-173	1.376541e-172
PDX-CDS#1/PDX-CDS#1-C	0.95	PDX-CDS#3/PDX-CDS#3-C	0.95	1	1
CDS#1/PDX-CDS#1	0.95	CDS#4/PDX-CDS#4	0.92	3.901132e-22	3.511019e-21
CDS#1/PDX-CDS#1	0.95	CDS#4/PDX-CDS#4-C	0.88	8.601183e-74	7.741065e-73
CDS#1/PDX-CDS#1	0.95	PDX-CDS#4/PDX-CDS#4-C	0.92	3.901132e-22	3.511019e-21
CDS#1/PDX-CDS#1-C	0.93	CDS#4/PDX-CDS#4	0.92	0.005708002	0.05137202
CDS#1/PDX-CDS#1-C	0.93	CDS#4/PDX-CDS#4-C	0.88	2.011544e-29	1.81039e-28
CDS#1/PDX-CDS#1-C	0.93	PDX-CDS#4/PDX-CDS#4-C	0.92	0.005708002	0.05137202
PDX-CDS#1/PDX-CDS#1-C	0.95	CDS#4/PDX-CDS#4	0.92	3.901132e-22	3.511019e-21
PDX-CDS#1/PDX-CDS#1-C	0.95	CDS#4/PDX-CDS#4-C	0.88	8.601183e-74	7.741065e-73
PDX-CDS#1/PDX-CDS#1-C	0.95	PDX-CDS#4/PDX-CDS#4-C	0.92	3.901132e-22	3.511019e-21
CDS#3/PDX-CDS#3	0.79	CDS#4/PDX-CDS#4	0.92	1.603017e-94	1.442715e-93
CDS#3/PDX-CDS#3	0.79	CDS#4/PDX-CDS#4-C	0.88	7.533332e-34	6.779999e-33
CDS#3/PDX-CDS#3	0.79	PDX-CDS#4/PDX-CDS#4-C	0.92	1.603017e-94	1.442715e-93
CDS#3/PDX-CDS#3-C	0.81	CDS#4/PDX-CDS#4	0.92	1.082479e-75	9.742311e-75
CDS#3/PDX-CDS#3-C	0.81	CDS#4/PDX-CDS#4-C	0.88	3.685744e-23	3.31717e-22
CDS#3/PDX-CDS#3-C	0.81	PDX-CDS#4/PDX-CDS#4-C	0.92	1.082479e-75	9.742311e-75
PDX-CDS#3/PDX-CDS#3-C	0.95	CDS#4/PDX-CDS#4	0.92	3.901132e-22	3.511019e-21
PDX-CDS#3/PDX-CDS#3-C	0.95	CDS#4/PDX-CDS#4-C	0.88	8.601183e-74	7.741065e-73
PDX-CDS#3/PDX-CDS#3-C	0.95	PDX-CDS#4/PDX-CDS#4-C	0.92	3.901132e-22	3.511019e-21
Paired comparisons based on 71 gene signature					
**Var1**	**rho_Var1**	**Var2**	**rho_Var2**	**p-value**	**Bonferroni Correction**
CDS#1/PDX-CDS#1	0.94	CDS#3/PDX-CDS#3	0.71	0.0000007000287	6.300258e-06
CDS#1/PDX-CDS#1	0.94	CDS#3/PDX-CDS#3-C	0.74	0.000004384242	3.945818e-05
CDS#1/PDX-CDS#1	0.94	PDX-CDS#3/PDX-CDS#3-C	0.92	0.3848785	0.9999
CDS#1/PDX-CDS#1-C	0.93	CDS#3/PDX-CDS#3	0.71	0.000006896242	6.206618e-05
CDS#1/PDX-CDS#1-C	0.93	CDS#3/PDX-CDS#3-C	0.74	0.00003662618	0.0003296356
CDS#1/PDX-CDS#1-C	0.93	PDX-CDS#3/PDX-CDS#3-C	0.92	0.6858797	0.9999
PDX-CDS#1/PDX-CDS#1-C	0.92	CDS#3/PDX-CDS#3	0.71	4.269073e-05	0.0003842166
PDX-CDS#1/PDX-CDS#1-C	0.92	CDS#3/PDX-CDS#3-C	0.74	0.0001966045	0.00176944
PDX-CDS#1/PDX-CDS#1-C	0.92	PDX-CDS#3/PDX-CDS#3-C	0.92	1	1
CDS#1/PDX-CDS#1	0.94	CDS#4/PDX-CDS#4	0.95	0.5846922	0.9999
CDS#1/PDX-CDS#1	0.94	CDS#4/PDX-CDS#4-C	0.90	0.1211317	0.9999
CDS#1/PDX-CDS#1	0.94	PDX-CDS#4/PDX-CDS#4-C	0.89	0.06528511	0.587566
CDS#1/PDX-CDS#1-C	0.93	CDS#4/PDX-CDS#4	0.95	0.3120004	0.9999
CDS#1/PDX-CDS#1-C	0.93	CDS#4/PDX-CDS#4-C	0.90	0.2776775	0.9999

CDS#1/PDX-CDS#1-C	0.93	PDX-CDS#4/PDX-CDS#4-C	0.89	0.167953	0.9999
PDX-CDS#1/PDX-CDS#1-C	0.92	CDS#4/PDX-CDS#4	0.95	0.1569259	0.9999
PDX-CDS#1/PDX-CDS#1-C	0.92	CDS#4/PDX-CDS#4-C	0.90	0.4958092	0.9999
PDX-CDS#1/PDX-CDS#1-C	0.92	PDX-CDS#4/PDX-CDS#4-C	0.89	0.3298787	0.9999
CDS#3/PDX-CDS#3	0.71	CDS#4/PDX-CDS#4	0.95	3.631408e-08	3.268267e-07
CDS#3/PDX-CDS#3	0.71	CDS#4/PDX-CDS#4-C	0.90	0.0006465045	0.00581854
CDS#3/PDX-CDS#3	0.71	PDX-CDS#4/PDX-CDS#4-C	0.89	0.001820489	0.0163844
CDS#3/PDX-CDS#3-C	0.74	CDS#4/PDX-CDS#4	0.95	2.764598e-07	2.488138e-06
CDS#3/PDX-CDS#3-C	0.74	CDS#4/PDX-CDS#4-C	0.90	0.002348234	0.02113411
CDS#3/PDX-CDS#3-C	0.74	PDX-CDS#4/PDX-CDS#4-C	0.89	0.00597807	0.05380263
PDX-CDS#3/PDX-CDS#3-C	0.92	CDS#4/PDX-CDS#4	0.95	0.1569259	0.9999
PDX-CDS#3/PDX-CDS#3-C	0.92	CDS#4/PDX-CDS#4-C	0.90	0.4958092	0.9999
PDX-CDS#3/PDX-CDS#3-C	0.92	PDX-CDS#4/PDX-CDS#4-C	0.89	0.3298787	0.9999

Supplementary Table S9: Drugs sensitivity in CDS and EWS PDX-derived cell lines.

**Drug**	**PDX-CDS #1-C**	**PDX-CDS #3-C**	**PDX-CDS #4-C**	**PDX-EWS #2-C**	**PDX-EWS #4-C**	**PDX-EWS #5-C**
**Doxorubicin (µM)**	0.5 ± 0.22	0.17 ± 0.07	0.17 ± 0.03	0.09 ± 0.03	0.03 ± 0.003	0.01 ± 0.0004
**Vincristine (µM)**	> 0.1	0.0007± 0.0002	0.006 ± 0.001	0.007 ± 0.002	0.001 ± 0.001	0.002 ± 0.0007
**Irinotecan (µM)**	4.4 ± 1.6	0.99 ± 0.13	4.1 ± 2	0.38 ± 0.16	0.009± 0.002	0.006 ± 0.004
**Ifosfamide (µM)**	3.34 ± 1.1	3.38 ± 1.91	27.3 ± 15.2	2.93 ± 1.02	2.24 ± 0.52	1.29 ± 0.26
**Etoposide (µM)**	15.12 ± 2.2	4.08 ± 1.35	1.3 ± 0.3	0.75 ± 0.29	0.09 ± 0.03	0.05 ± 0.005

Abbreviations: CDS, CIC-DUX4 sarcoma; EW, Ewing sarcoma; PDX, patient-derived xenograft.

IC_{50} values after 72 hours of treatment are reported.
