# Supplementary material for: Integrated Molecular Characterization of Patient-Derived Models Reveals Therapeutic Strategies for Treating CIC-DUX4 Sarcoma
Source: Cancer Res. 2021 Dec 13;82(4):708–20. doi: 10.1158/0008-5472.CAN-21-1222 (PMC9359717; doi:10.1158/0008-5472.CAN-21-1222)
Supplement: Supplementary Data [file can-21-1222_supplementary_methods_and_figures_supp1-13.pdf]

Supplementary Data

Integrated molecular characterization of patient-derived models reveals therapeutic strategies for treating CIC-DUX4 sarcoma

Marianna Carrabotta, Maria Antonella Laginestra, Giorgio Durante, Caterina Mancarella, Lorena Landuzzi, Alessandro Parra, Francesca Ruzzi, Lisa Toracchio, Alessandra De Feo, Veronica Giusti, Michela Pasello, Alberto Righi, Pier-Luigi Lollini, Emanuela Palmerini, Davide Maria Donati, Maria Cristina Manara* and Katia Scotlandi*.

*Corresponding authors. Email: mariacristina.manara@ior.it; katia.scotlandi@ior.it

1. Supplementary Materials and Methods

2. **Supplementary Fig. S1**
Unsupervised and supervised hierarchical clustering (HC)

3. **Supplementary Fig. S2**
Gene Set Enrichment Analysis

4. **Supplementary Fig. S3-S**4
Unsupervised hierarchical clustering: 3,179 or 537 differentially expressed genes between CDS and EWS patients were applied to an *in-silico* dataset of other human fusion-driven sarcomas and normal mesenchymal stem cells

5. **Supplementary Fig. S5**
Unsupervised hierarchical clustering: 71 differentially expressed genes between CDS and EWS patients were applied to an *in-silico* dataset of other human fusion-driven sarcomas and normal mesenchymal stem cells

6. Supplementary Fig. S6
Coefficient of Variation compute on 3,179 genes in the two groups (CDS and EWS)

7**. Supplementary Fig. S7**
MetaCore analysis

8. **Supplementary Fig. S8**
Immunostaining of CDS human tumor samples and corresponding PDXs

9. **Supplementary Fig. S9**
Evidence of CIC-DUX4 fusion transcripts in CDS patients, PDXs and PDX-derived cell lines

10. **Supplementary Fig. S10**
RNA interference silencing of HMGA2 in PDX-CDS#3-C cells

11. **Supplementary Fig. S11**
Akt signaling mediators in PDX-CDS-C#4 cells treated or not with the anti-IGF1R hAb AVE 1642

12. **Supplementary Fig. S12**
Isobologram schematic representation of the effects of combined NVP-BEZ235 and trabectedin treatment in PDX-CDS#4-C and PDX-CDS#3-C cells

13. **Supplementary Fig. S13**
Immunohistochemical evaluation of p-Akt, p-mTOR, and p-S6 in xenografts after s.c. injection of PDX-CDS#4 cells treated with trabectedin, NVP-BEZ235 or the combination of the two drugs

Supplementary Materials and Methods

RNA-seq library preparation

All patients had a diagnosis of CDS or EWS made on representative specimens based on histologic, cytologic, and immunohistochemistry (IHC) features as well as the molecular presence of the chimeric product.

RNA-seq data analysis

FASTQ files were quality controlled using FastQC Version 0.11.9 (http://www.bioinformatics.babraham.ac.uk/projects/fastqc/; RRID:SCR_014583) and analyzed using RNA Express BaseSpace App (Illumina; RRID:SCR_011881). Paired end reads were aligned to the human reference sequence GRCh37 by STAR alignment (RRID:SCR_004463) (1). Then, HT-Seq Counts (RRID:SCR_011867) and Deseq2 (RRID:SCR_015687) were used to assign aligned reads to genes and to perform differential gene expression (computed on raw counts) (2,3).

Raw counts were normalized according to the library size to obtain transcripts per million (TPM), and 14,334 genes were used to generate unsupervised hierarchical clustering (HC) and 3D principal component analysis (PCA). Unsupervised HC was generated using the Hclust R function (RRID:SCR_009154) based on the Ward.D2 method and Euclidean distance as a measure of similarity (R package stats v3.6.2) (RRID: SCR_000432). PCA was conducted using the pricomp R function (4) and visualized by the 3D visualization device system RGL (R package version 0.100.54; https://CRAN.R-project.org/package=rgl).

Starting from the Deseq2 output, we performed several steps of selection and filtration of data; in particular, the output was first selected for the status (column of Deseq2 output) of the genes, which included outliers (genes in which the result was strongly affected by a single outlier replicate), low genes (genes with a low average expression across samples with a mean count across all samples less than 10), and OK status. Therefore, only the genes with an output corresponding to OK status

were selected and the whole list of these genes was filtered for an Benjamini-Hochberg (BH) adjusted p-value <0.05.

Then, from this gene list, we analyzed the single gene level counts in the two groups (EWS and CDS) by selecting genes with a mean raw count >5 for each group. In addition, we specifically analyzed the selected genes in the context of their expression. We performed a coefficient of variation analysis (CV=Standard Deviation/Mean) for the selected genes in both the two sample groups (CDS and EWS) and considered a CV threshold ≤0.25 (CV<0.1 very good; 0.1-0.2 good; 0.2-0.3 acceptable) (5). This analysis was computed on regularized logarithm transformation (rlog) of raw count (3).

The selected signatures were used for Spearman's correlation analysis among CDS patients, PDXs and corresponding PDX-derived cell lines and we calculated the confidence interval ($CI_{95\%}$) that provides a measure of the overlapping among the correlation coefficients (6). Furthermore, we applied statistical significance tests to assess the comparison of two correlation coefficients either in intra- and inter- CDS/PDX/PDX derived cell line groups (7,8).

Heatmaps and correlograms were generated using the ComplexHeatmap (RRID:SCR_017270) and CorrPlot R packages, respectively (9).

Briefly, for heatmap generation we considered rlog (regularized logarithm) of raw counts, and then we applied the Z-score scaling method for visualization of differentially expressed genes between CDS and EWS patients. All analyses were performed using R version 4.0.3 (Bioconductor, http://www.bioconductor.org/; RRID:SCR_006442).

Functional analysis was performed using gene set enrichment analysis (GSEA; RRID:SCR_003199). The Molecular Signature Database (MsigDB) c2.all.v7.0.symbols.gmt signature (RRID:SCR_016863) was used and gene sets with between 15 and 200 members were considered. GSEA was performed using GSEA-pre-ranked module against our ranked list of 3,179 genes, to determine whether a priori defined sets of genes show significant enrichment at either end of the ranking (top or bottom ranks). In particular, we performed the following steps: 1) we computed

differential expression analysis using DeSeq2; and 2) we ranked our gene list (from most upregulated to most downregulated genes), choosing as ranking metric the $\log_2$FC (fold change) (10,11) . The most relevant gene sets were selected considering a normalized enrichment score (NES) ≥2.5 and a false discovery rate (FDR) ≤ 0.001. After selecting the most relevant gene sets we performed leading-edge analysis to investigate key genes related to the transcriptomic changes of CDS samples. In particular, the leading edge analysis is determined from the enrichment score (ES), which is defined as the maximum deviation from zero (10) . First, to start with the leading edge analysis, we selected the gene sets that were to be compared from, by ranking the gene sets based on the FDR≤ 0.001. and NES≥2.5 as cutoff (12) . The web-based software MetaCore (GeneGo, Thomson Reuters, RRID:SCR_008125) was used to put the genes resulting from leading edge analysis in the context of a protein-protein interaction network. Starting from the list of 71 core enriched genes, we used the "direct interaction" algorithm to evaluate the protein-protein interaction of these 71 genes to build a network. Then, we used only the "objects" (genes) in the original list; no other "objects" from the MetaCore database were added. Only the "objects" in the list that can directly interact with each other resulted in a "link" between two network objects.

To validate the gene signatures characteristic of CDS, we considered a published microarray-based gene expression data set of 14 CDS (GSE60740), 7 EWSR1-NFATc2 (GSE60740), 27 EWS (E-MEXP-1142), 8 monophasic synovial sarcomas (MSS, E-MEXP-353), 6 myxoid liposarcomas (MLS, E-MEXP-353), 4 rhabdomyosarcoma (ARMS, E-MEXP-353) patient-derived tumors and 17 normal mesenchymal stem cells (MSC)(GSE7888).

Raw CEL file data were normalized using robust multi-array average normalization (RMA) and $\log_2$ transformed. To reduce the variability across different data sets, we performed batch effect correction using the removeBatchEffect function from the limma R package (11). Unsupervised HC was adopted to test the gene signature, and Z-scores of $\log_2$ transformed expression values were displayed by ComplexHeatmap R package (RRID:SCR_017270) (9).

Establishment of CDS PDXs and PDX-derived cell lines

To generate PDXs, a fresh tumor specimen approximately 4 mm^3 in volume was implanted subcutaneously (s.c.) at the level of interscapular brown fat into 5–11-week-old immunodeficient NOD SCID gamma (NSG) mice (Charles River; RRID:IMSR_ARC:NSG) within an average of 1–2 hours following resection from the patient. Tumor growth was checked at least twice weekly until it reached the endpoint volume of 2.5 cm^3, at which point the mouse was sacrificed by inhalation of the CO_2 and cervical dislocation. Accurate necropsy was performed and lung metastases were outlined by the intratracheal injection of black India ink. The lungs were fixed in Fekete’s solution and metastases were counted under a stereoscope. The tumor was resected and tumor fragments were implanted in NSG mice to produce subsequent PDX passages, as previously described by Nanni *et al.* (13). The remaining fragments were finely minced with scissors and either used for histopathological and molecular analyses or seeded in flasks with complete growth medium to obtain a PDX-derived cell line.

All PDX-derived cell lines were authenticated by DNA fingerprinting (AMEL, D3S1358, TH01, D21S11, D18S51, D10S1248, D1S1656, D2S1338, D16S539, D22S1045, VWA, D8S1179, FGA, D2S441, D12S391, D19S433 and SE33; last control July 2020; POWERPLEX ESX 17 Fast System, Promega) compared to the profile of the original surgical specimen and of the PDX when appropriate. Cell lines were immediately expanded to generate liquid nitrogen stocks and were never passaged for more than one month after thawing to avoid genetic or phenotypic modifications due to culture conditions. Cells were maintained in Iscove's modified Dulbecco's medium (IMDM; ECB2072L, Euroclone) supplemented with 10% heat-inactivated FBS (ECS0180L, EuroClone), 100 U/mL penicillin and 100 mg/mL streptomycin (P0781-100ML, Merck) in a 37°C humidified environment at 5% CO_2. All cell lines were tested for mycoplasma-contamination by using MycoAlert Mycoplasma detection kit (LT07-418, Lonza) every 3 months.

Assessment of CIC-DUX4 rearrangements and contamination of murine fibroblasts

For the identification of CIC-DUX4 rearrangements, 500 ng of total RNA was reverse transcribed and amplified by PCR (primer set: Fw: 5'-GAGGACGTGCTTGGGGAGCTAGAGT-3', 2R2 Rv: 5'-CGCTGTGTGGAGTCTCTCACCG-3', ex2 Rv: 5'- CCGCGGAGTGGAGTCTCTCACCG-3'). A negative control and a 100 bp DNA ladder (G210A, Promega) were included in each RT- PCR assay (Supplementary Fig. S8).

All cell lines were qPCR screened for the contamination of murine fibroblasts; briefly, gDNA was extracted by proteinase K digestion and silica column purification from the newly established PDX-derived cell line using PureLink Genomic DNA minikit (K1820-01, Thermo Fisher Scientific). Forty nanograms of total gDNA were screened for the amplification of two murine genes and a human (human *ACTB* and murine *Gapdh* and *Actb*). The quantification of the human to mouse ratio was achieved through the interpolation to a relative standard curve for each gene to verify the exclusive presence of human cells.

qRT-PCR analysis

Predesigned TaqMan probes (Thermo Fisher Scientific) were used to determine the expression levels of *HMGA1* (Hs00431242_m1), *HMGA2* (Hs04397751_m1), *IGF2BP2* (Hs00538956_m1), and *IGF2BP3* (Hs00559907_g1); a primer sets for *IGF2* (Fw: 5'-GACCGCGGCTTCTACTTCAG-3', Rv: 5'-AAGAACTTGCCCACGGGGTAT-3') were used for SYBR Green quantification and a primer pairs for the reference gene *GAPDH* were used as previously reported (14). Total RNA from primary cultures of human mesenchymal stem cells (hMSCs) obtained during surgery was used as interplate calibrator.

Western blotting

Harvested cells were rinsed in 1X PBS and lysed in phosphoprotein extraction buffer supplemented with protease-phosphatase cocktail inhibitor (P8340, Merck). Western blotting was executed according to standard procedures. Membranes were incubated overnight with the following primary antibodies: anti-HMGA2 (rabbit polyclonal, GTX100519, 1:4000, GeneTex, RRID:AB_1950498); anti-IGF2BP2 (rabbit, polyclonal RN008P, dilution 1:5000, MBL International, RRID:AB_1570641); anti-IGF2BP3 (polyclonal, rabbit, RN009P, dilution 1:5000, MBL International, RRID:AB_1570642); anti-IGF2 (rabbit, polyclonal, ab9574, dilution 1:1000, Abcam, RRID:AB_308725); anti-IGF1Rβ (rabbit, polyclonal, sc-713, dilution 1:1000, Santa Cruz Biotechnology, RRID:AB_671792); anti-GAPDH (14C10, rabbit, 2118, dilution 1:5000, Cell Signaling Technology, RRID:AB_10693448); anti-phospho-AKT (Ser473) (736E11, rabbit, #3787, dilution 1:1000, Cell Signaling Technology, RRID:AB_331170), anti-AKT (rabbit, polyclonal, #9272, dilution 1:3000, Cell Signaling Technology, RRID:AB_329827), anti-phospho-mTOR (Ser2448) (rabbit, polyclonal, #2971S, dilution 1:1000, Cell Signaling Technology, RRID:AB_330970), anti-mTOR (rabbit, polyclonal, #2972, dilution 1:2000, Cell Signaling Technology, RRID:AB_330978), anti-phospho-p44/42 MAPK (Erk1/2) (Thr202/Tyr204) (rabbit, 197G2, #4377, dilution 1:1000, Cell Signaling Technology, RRID:AB_331775), anti-p44/42 MAPK (Erk1/2) (rabbit, polyclonal, #9102, dilution 1:5000, Cell Signaling Technology, RRID:AB_330744), anti-phospho S6 ribosomal protein (Ser240/244) (rabbit, polyclonal, #2215, dilution 1;2000, Cell Signaling Technologies, RRID:AB_331682), anti-S6 ribosomal protein (rabbit, 54D2, #2317, dilution 1;2000, Cell Signaling Technologies, RRID:AB_2238583). Donkey Anti-Rabbit IgG, Whole Ab ECL Antibody, HRP Conjugated (NA934, dilution 1:1000-1:5000, GeHealthcare, RRID:AB_772206) was used as secondary antibody.

Preclinical *in vitro* experiments

In vitro drug sensitivity was assessed using the TACS® MTT Cell Proliferation Assay kit (#4890/25/K, Trevigen) according to the manufacturer's instructions. Cells were plated into 96-well plates (40,000 cells/well) in standard medium and cultured for 24 hours, before they were treated with the chemicals listed in the paper for 72 hours. Where applicable, cells were also treated with dimethyl sulfoxide (DMSO, #D2650, Merck) as a control to a final concentration <0.001%, which had no effect on cell growth. For the combination treatments, cells were sequentially administered trabectedin for 24 hours (dosage: 0.03 nmol/L for PDX-CDS#4-C and 0.1 nmol/L for PDX-CDS#3-C corresponding to doses that cause 20-30% growth inhibition, respectively), followed by NVP-BEZ235 (dosage: 0.01-0.03-0.06-0.1-0.3-1 µmol/L) for additional 48 hours.

For basal gene and protein expression analysis, $1x10^6$ cells were grown in standard medium for 48 hours and processed for qRT-PCR and western blotting, as described above.

To evaluate the molecular effects of IGF2, PDX-CDS and PDX-EWS cells were seeded (1×10^6 cells) in IMDM supplemented with 10% FBS. Cells were serum-starved for 24 hours in IMDM containing 1% FBS before they were exposed to IGF2 (50 ng/mL, # I2526, Merck Life Science) for 24 hours. Harvested cells were used for the preparation of cell lysates for western blot.

To evaluate the molecular effects of trabectedin, semiconfluent cells were treated with trabectedin (3 nmol/L) for 3-6 hours for qRT-PCR and for 24-48 hours for protein analysis.

Knockdown of CIC-DUX4 was achieved using CIC-specific siRNAs from Qiagen (SI04368469|S1 - Hs_CIC_8 FlexiTube siRNA SI04275656|S1 and Hs_CIC_6 FlexiTube siRNA). AllStars Negative Control siRNA (5 nmol/L) (Cat. No. 1027280, Qiagen) was used as a control. A combination of both siRNAs (20 nmol/L each) was transfected into PDX-CDS#4-C cells using TransIT-X2 (MIR6000, Mirus) in accordance with the manufacturers' protocol. Cells were harvested for qRT-PCR analysis 72 hours after transfection. Silencing was checked by PCR (primer

set: Fw: 5’-GAGGACGTGCTTGGGGAGCTAGAGT-3’, ex2 Rv: 5’-CCGCGGAGTGGAGTCTCTCACCG-3’).

Transient silencing of HMGA2 was performed using short interfering RNA (siRNA) SMART POOL siGENOME_siRNA (M-013495-02-0020, GE Healthcare Dharmacon). As control, siGENOME_nontargeting siRNA was employed (D-001206-13-05, GE Healthcare Dharmacon). siRNAs (40nmol/L) were transfected into PDX-CDS#3-C cells using TransIT-X2 in accordance with the manufacturers’ protocol. Cells were harvested for qRT-PCR and protein analysis 120 hours after transfection.

To evaluate the impact of trabectedin in combination with NVP-BEZ235 on the phosphorylation status of the IGF1R/AKT pathway, semiconfluent cells were treated with trabectedin (3 nmol/L) for 24 hours and then with NVP-BEZ235 (dosage: 30, 100, 300 nmol/L) for an additional 24 hours.

To evaluate the impact of AVE 1642, semiconfluent cells were treated with AVE 1642 (1 µg/mL) for 1-24 hours. Harvested cells were used for preparation of cell lysates for western blot.

Pharmacological *in vivo* experiments

The treatment groups, both after s.c. and i.v. injection of 2×10^6 PDX-CDS#4-C cells, were as follows (5-7 mice per group): trabectedin, 0.15 mg/kg intravenously (i.v) at day 1 of treatment; NVP-BEZ235 25 mg/kg per os (p.o.) dissolved in NMP 10% (1-methyl-2-pyrrolidone, 328634, Merck)/PEG300 90% (202371, Merck) daily, 5 days/week, for 3 and 5 weeks, respectively; NVP-BEZ235 p.o. in combination with trabectedin i.v. following the time schedule mentioned above; the control group was not treated.

Tumor size was measure using calipers twice a week and tumor volumes were calculated according to the formula $\pi[\sqrt{(a\times b)}]^3/12$, where a=maximal tumor diameter and b=tumor diameter perpendicular to a. Mice developing s.c. tumors were sacrificed at 36 days after cell injection or for ethical reasons, when the tumors reached a volume of 2.5 cm^3.

Mice developing experimental metastases were sacrificed at 6 weeks from i.v. cell injection when very initial signs of metastatic growth appeared in the control group not receiving any treatment. The mice were subjected to an accurate necropsy. The lungs were perfused with black India ink;lung and liver metastases were counted using a dissection microscope, and any other metastatic lesion in other sites (mainly interscapular adipose tissue and lymph nodes) was recorded, collected and fixed in a 10% buffered formalin solution (HT501128, Merck).

Supplementary Fig. S1

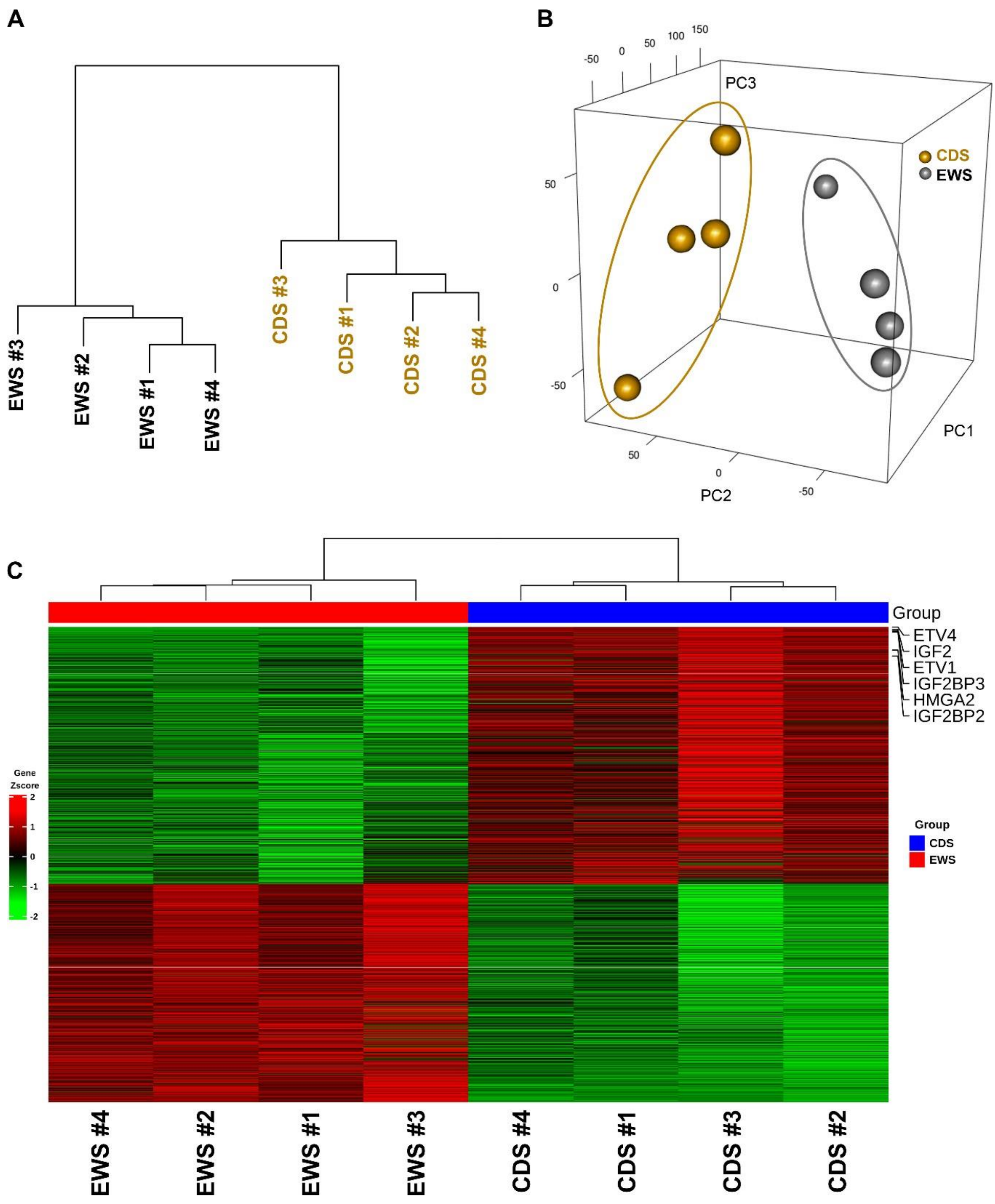

Unsupervised and supervised hierarchical clustering (HC)

(A) HC comparing CDS and EWS patient tumor samples. HC was generated using WARD.D2 method and Euclidean distance as a measure of similarity. **(B)** PCA plot shows the different transcriptomes between the CDS and EWS groups. In particular, the percentage of variance explained by the 3 main components was 78.8% (component 1, x-axis: 43.7%; component 2, y-axis: 25.4%;

component 3, z-axis: 9.7%). **(C)** 3,179 genes were differentially expressed between CIC-DUX4 *versus* Ewing sarcoma patients. In the matrix, each row represents a gene and each column represents a sample. The color scale illustrates the relative expression level of a gene across all samples: red represents an expression level above the mean, and green represents expression lower than the mean.

Supplementary Fig. S2

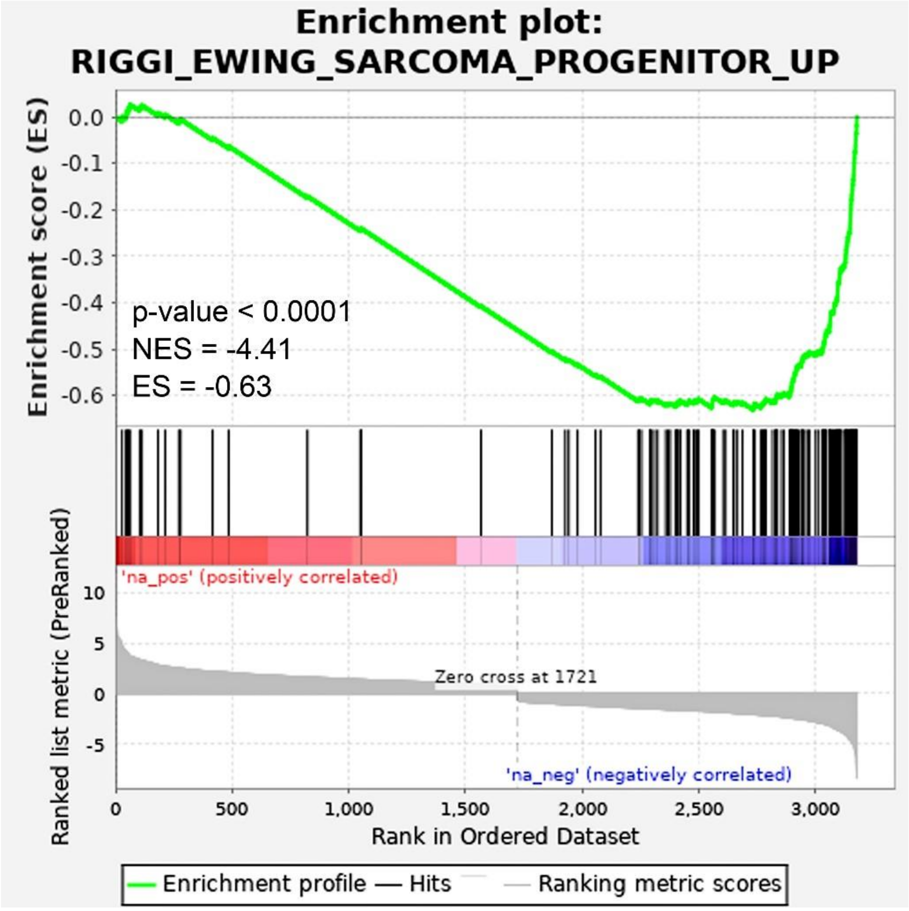

Gene Set Enrichment Analysis

GSEA revealed that the CIC-DUX4 transcriptional profile negatively correlated with the gene signature upregulated in Ewing sarcoma previously reported by Riggi *et al.* (15) . In the enrichment plot, the x-axis shows the rank order of genes from the most upregulated to the most downregulated between CIC-DUX4 and Ewing sarcoma samples. The vertical black line indicates the position of the enriched gene (Hit) comprising the gene set. The graph on the bottom shows the ranked list metric (signal-to-noise ratio) for each gene as a function of the rank in the ordered dataset.

Supplementary Fig. S3

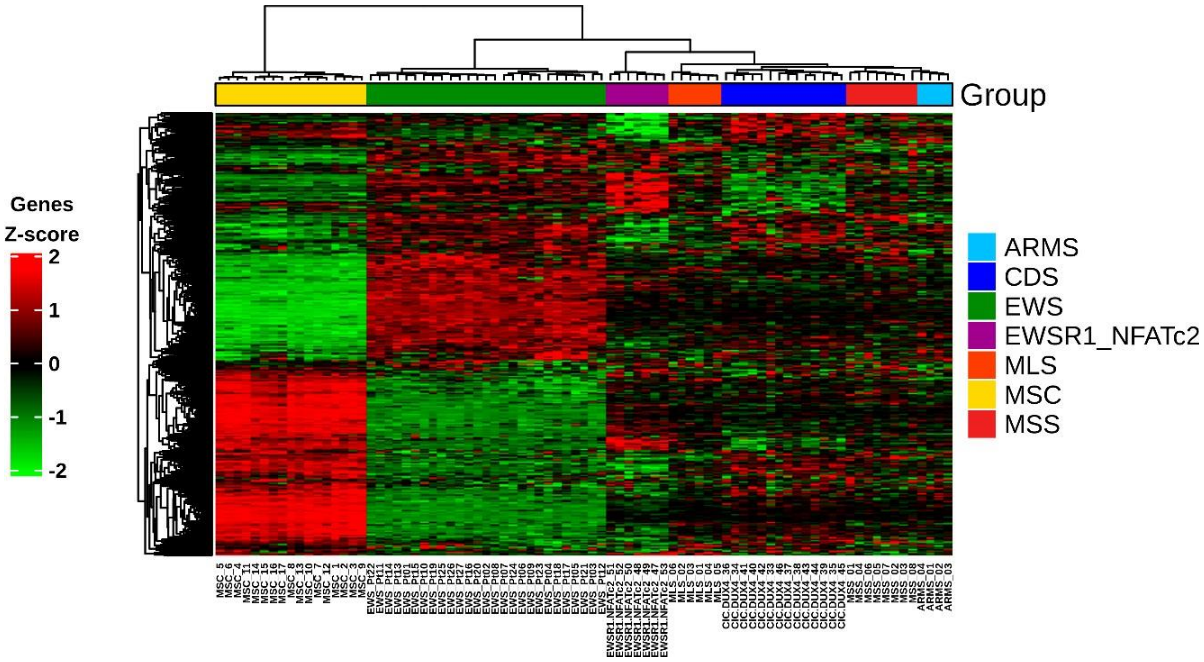

Supplementary Fig. S4

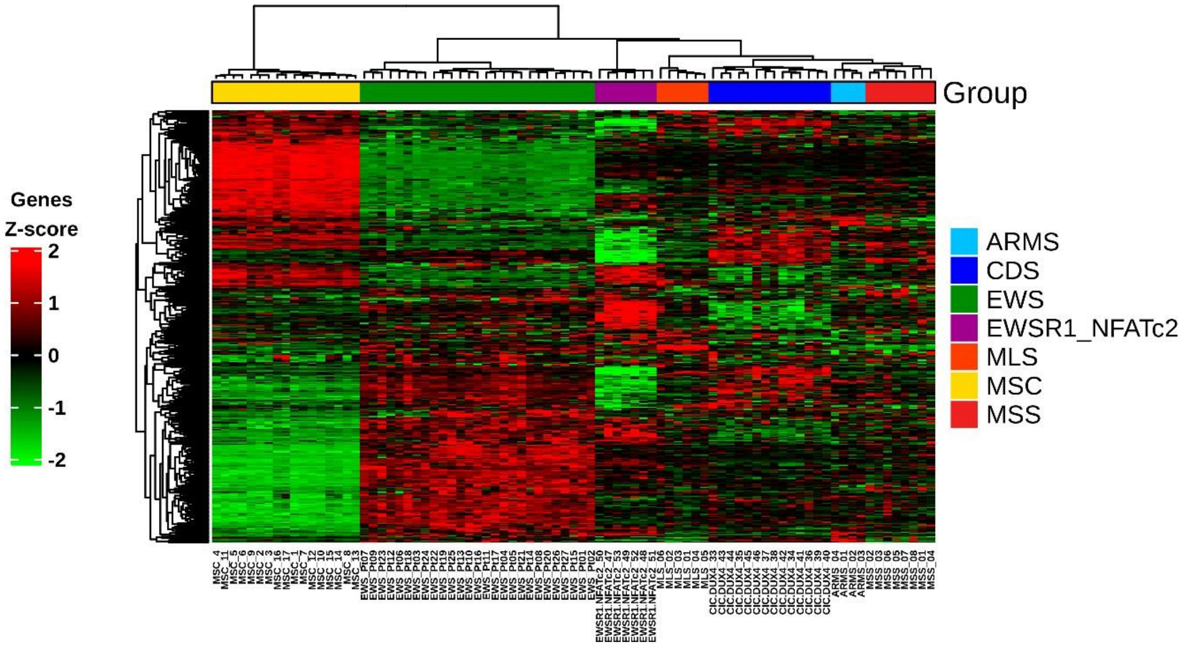

Supplementary Fig. S3-S4

Unsupervised hierarchical clustering: 3,179 and 537 gene signatures differentially expressed between CDS and EWS patients were applied to an *in silico* dataset of 14 CIC-DUX4 (CDS), 7 EWSR1-NFATc2, 27 Ewing (EWS), 8 monophasic synovial sarcomas (MSS), 6 myxoid liposarcomas (MLS), 4 rhabdomyosarcoma (ARMS) patient-derived tumors and 17 normal mesenchymal stem cells.

The hierarchical clustering algorithm showed a distinct cluster of normal mesenchymal stem cells from the other sarcoma tumors, and specifically, the CDS group presented a distinct cloud with respect to the other histotypes. In the matrix, each row represents a gene and each column represents a sample. The color scale illustrates the relative expression level of a gene across all samples: red represents an expression level above the mean and green represents expression lower than the mean.

Supplementary Fig. S5

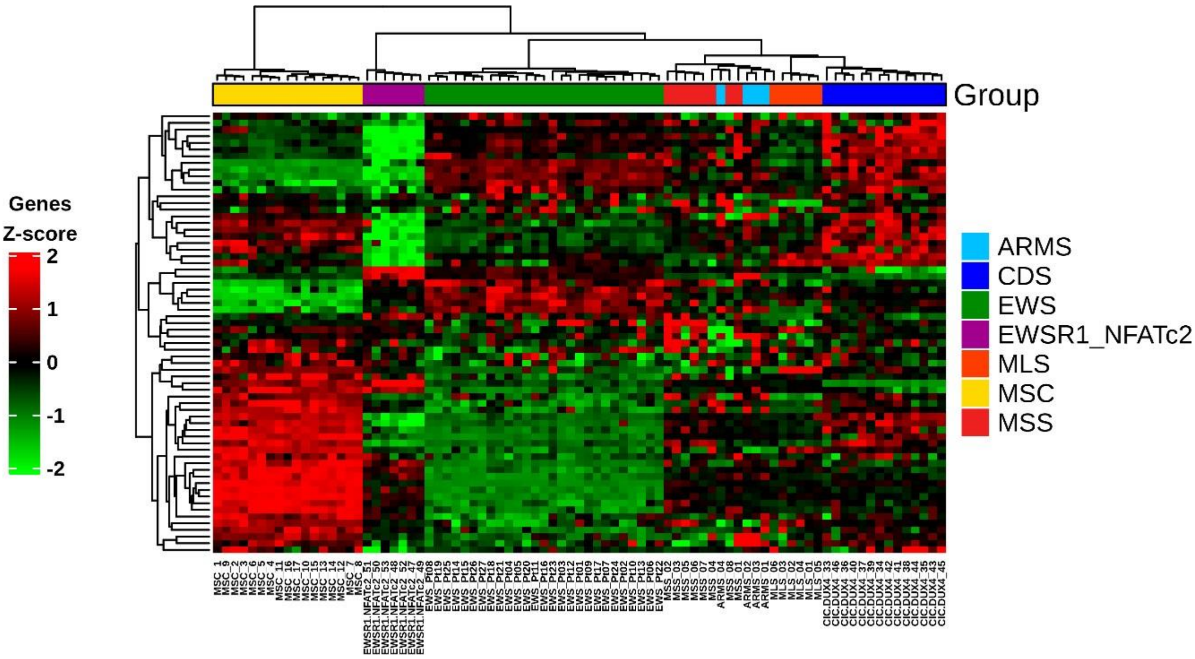

Unsupervised hierarchical clustering: 71 differentially expressed genes between CDS and EWS patients were applied to an *in silico* dataset of 14 CIC-DUX4 (CDS), 7 EWSR1-NFATc2, 27 Ewing (EWS), 8 monophasic synovial sarcomas (MSS), 6 myxoid liposarcomas (MLS), 4 rhabdomyosarcoma (ARMS) patient-derived tumors and 17 normal mesenchymal stem cells.

A hierarchical clustering algorithm showed a distinct cluster of CDS from other sarcoma tumors and mesenchymal stem cells, when the 71 enriched gene signature was applied. In the matrix, each row represents a gene and each column represents a sample. The color scale illustrates the relative expression level of a gene across all samples: red represents an expression level above the mean and green represents expression below the mean.

Supplementary Fig. S6

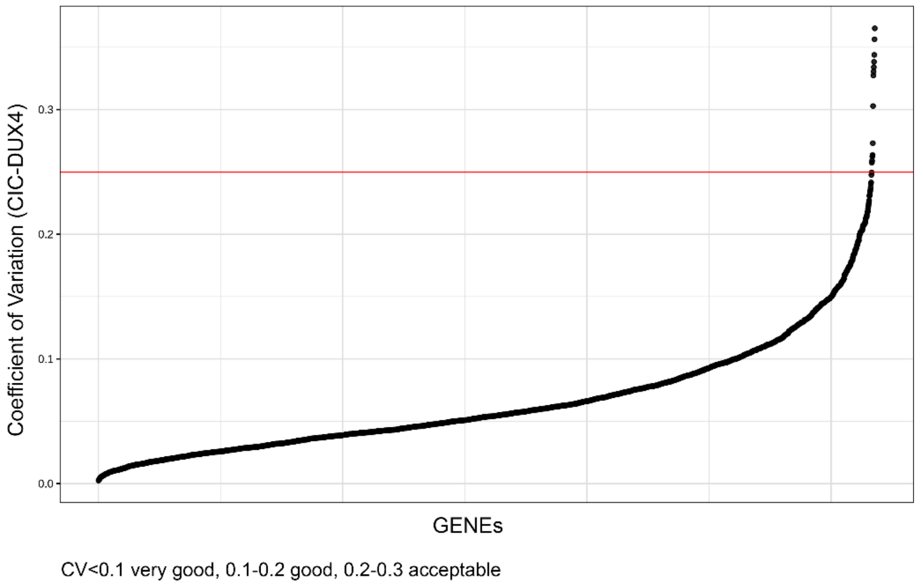

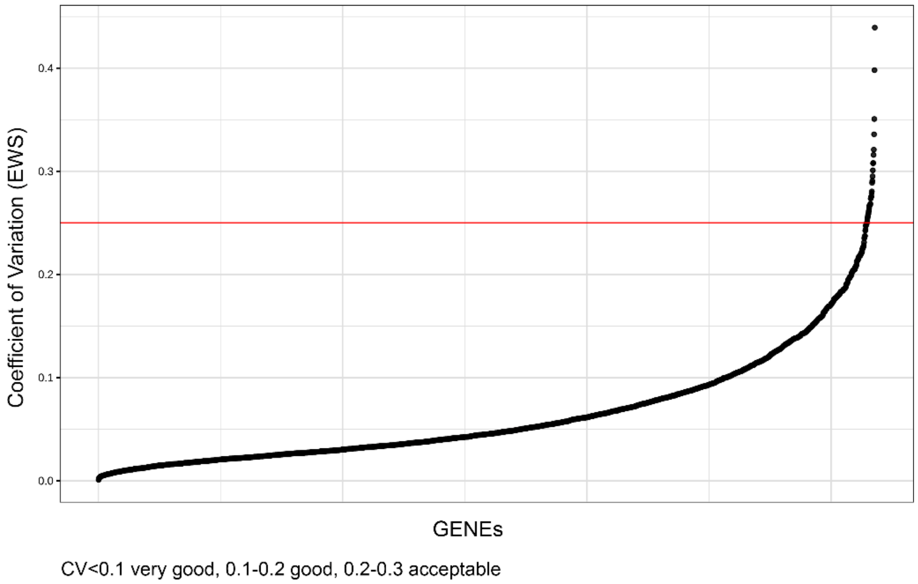

Coefficient of Variation performed on rlog raw count to evaluate the dispersion level of 3,179 expression gene values around the mean in the two group CDS and EWS. The genes showing a CV≥0.25 are reported below for the two groups.

CDS: *DGCR5, SIGLEC11, BMP7, PPP1R1B, ABCC8, ZNF99, NAT8L, KIF1A, PTGER2, COL22A1, AHRR, LRRC2, GGTA1P*.

EWS: *KIAA2022, NDNF, ADCY2, GCNT4, ZFPM2, ZIC2, NWD1, HLF, RNF182, LY86, SLC35F1, PPP1R1B, LTK, AR, CCRL2, FLRT1, LRRC10B, KIF4B, MNDA, CADPS, ABCC8, CCL3, FNDC5, BMP7, EPDR1, ETV7, DLX3, FCGR2B, PAX3, TTLL7, TCTEX1D1, CABP1, NAT8L*

Supplementary Fig. S7

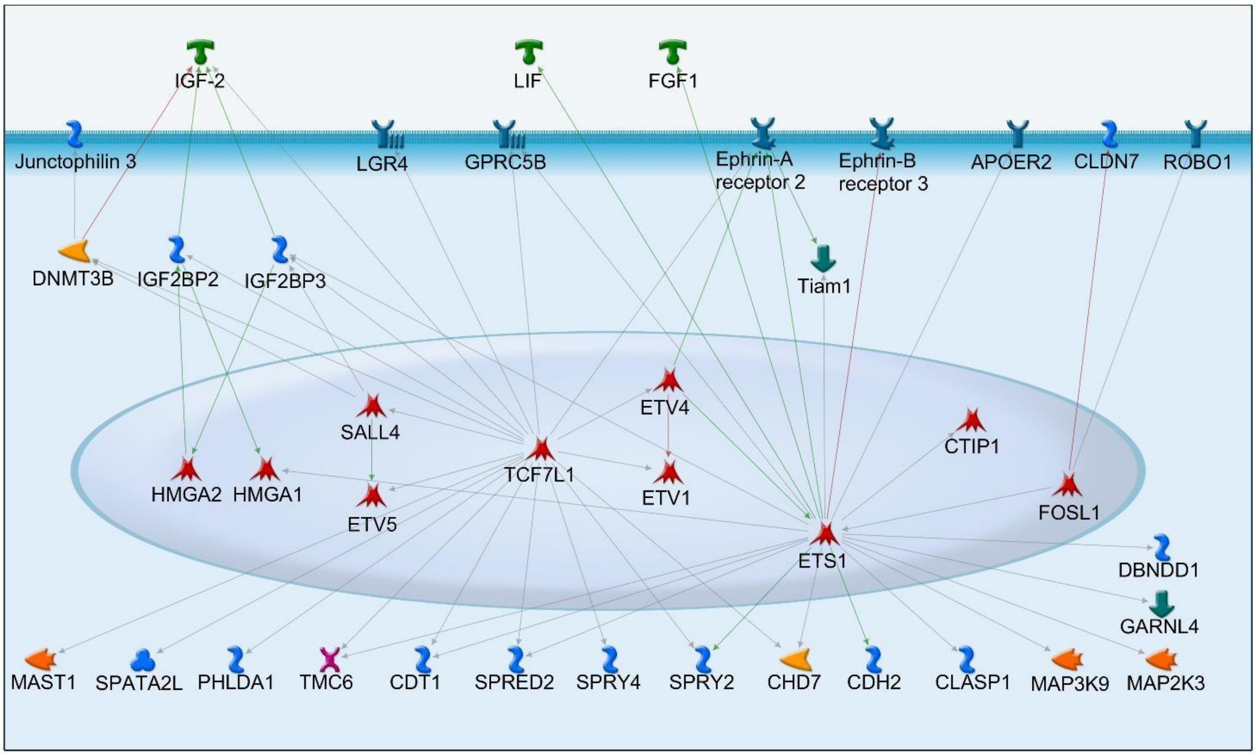

MetaCore analysis

The network was built starting from a subset of 71 DEGs retrieved from leading-edge analysis using the “direct interaction” algorithm. The resulting network map was graphically refined with GeneGo Pathway map creator software and genes with no interaction were omitted

Supplementary Fig. S8

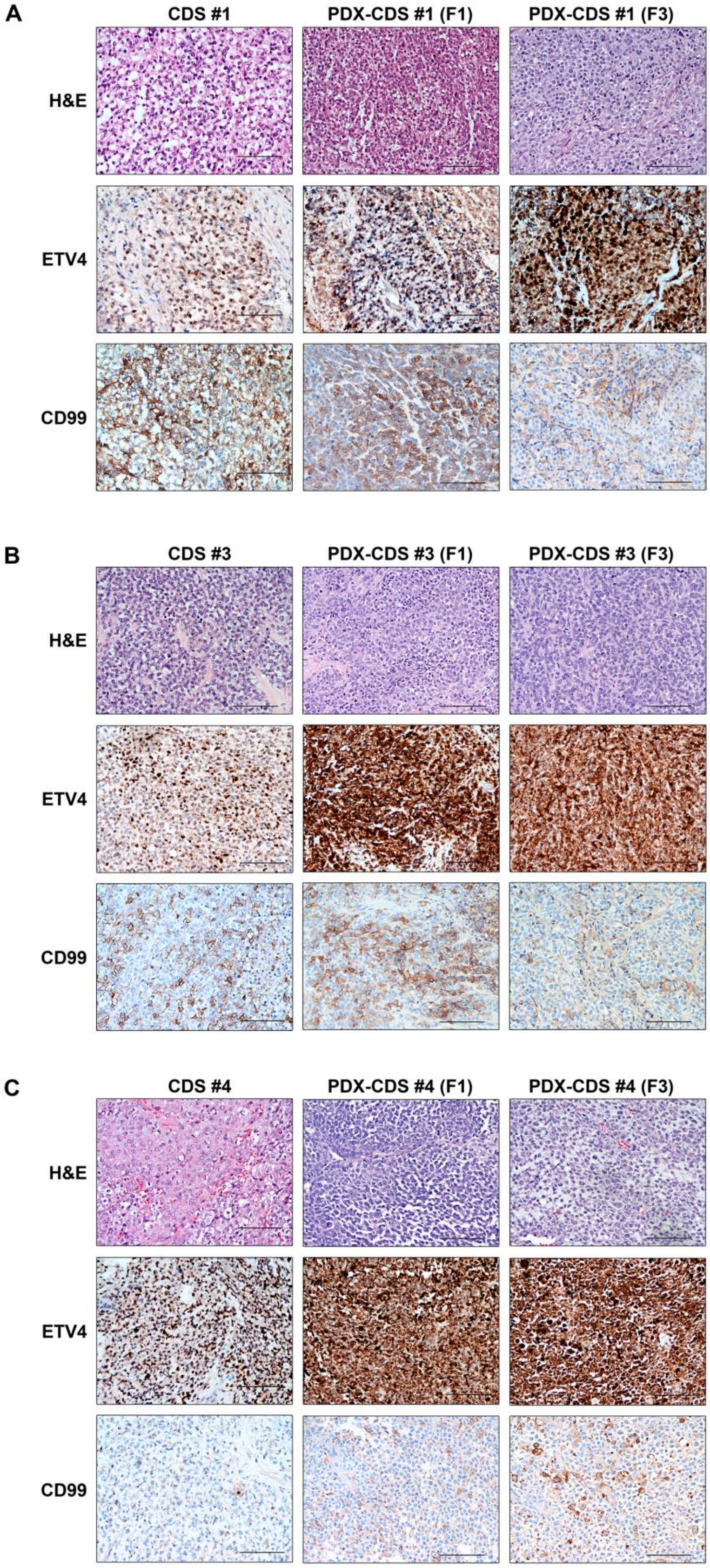

Immunostaining of CDS human tumor samples and corresponding PDX

Histologic and immunohistochemical features of patients' CDS#1 (top), CDS#3 (middle), CDS#4 (bottom) and corresponding PDX at different *in vivo* passages. Sections were stained with hematoxylin and eosin (H&E) or with antibodies against CDS biomarkers CD99 and ETV4. Bar: 100 µm.

Supplementary Fig. S9

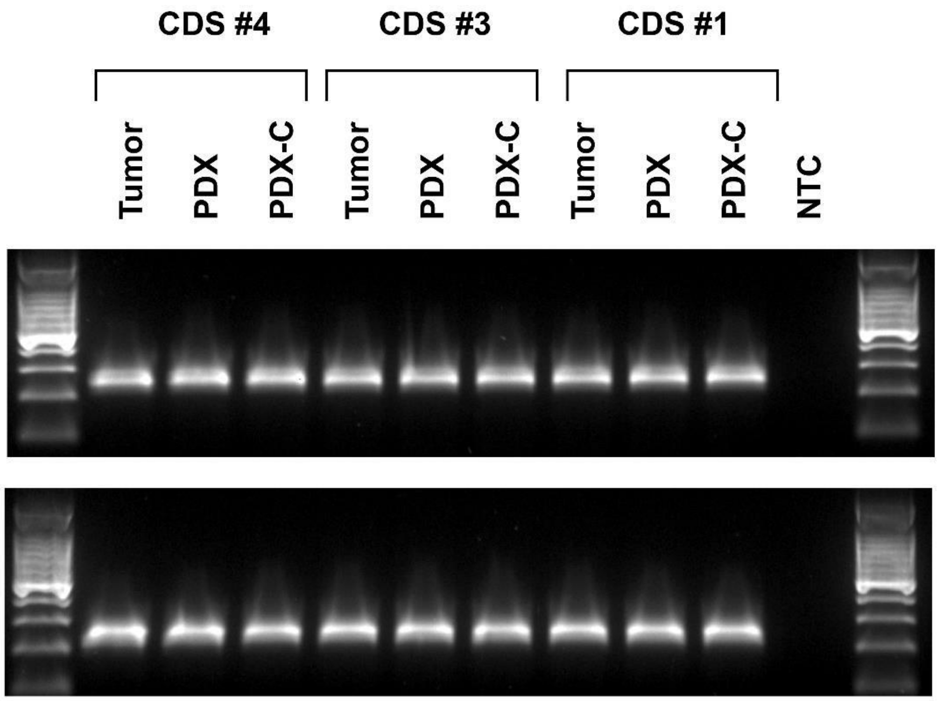

Evidence of CIC-DUX4 fusion transcript in CDS patients, PDXs and PDX-derived cell lines by RT-PCR using two distinct primers as reported in Supplementary Materials and Methods. Agarose gel electrophoresis images of a 233-base fusion transcript are shown. No-template controls (NTC) and 100bp marker are included.

Supplementary Fig. S10

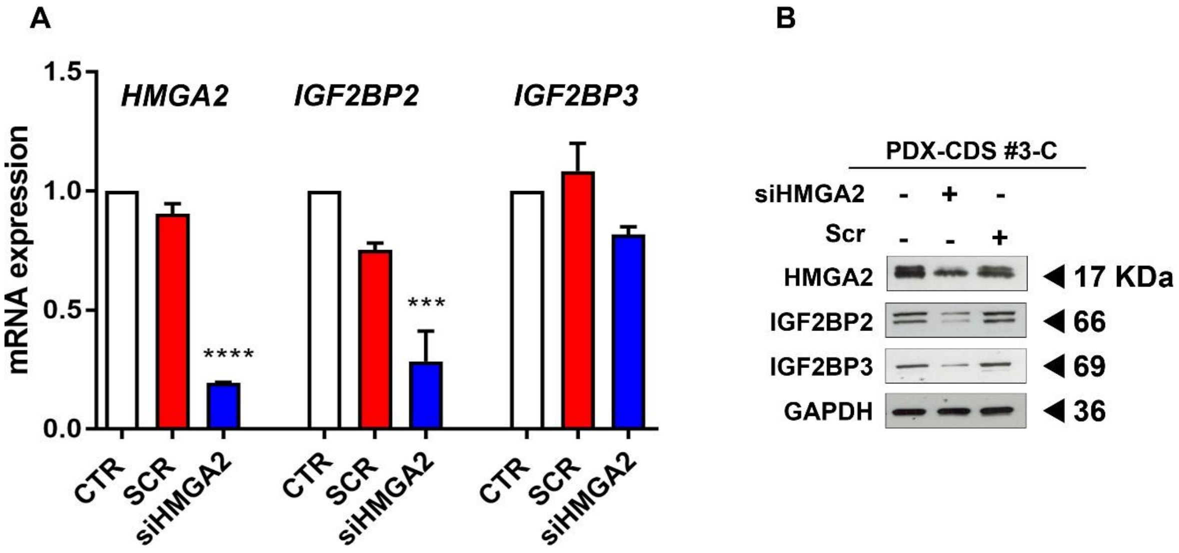

RNA interference silencing of HMGA2 in PDX-CDS#3-C cells.

qRT-PCR (**A**) and western blot (**B**) analysis of HMGA2, IGF2BP2, IGF2BP3analyses were performed after 120 hours of transfection with siHMGA2 (40 nmol) or scrambled control siRNA (40 nmol). Two independent biological replicates were performed. One representative immunoblot is shown. GAPDH was used as reference gene. For qRT-PCR, the data represent the mean ± SE. *** p < 0.001; **** p < 0.0001, one-way ANOVA.

Supplementary Fig. S11

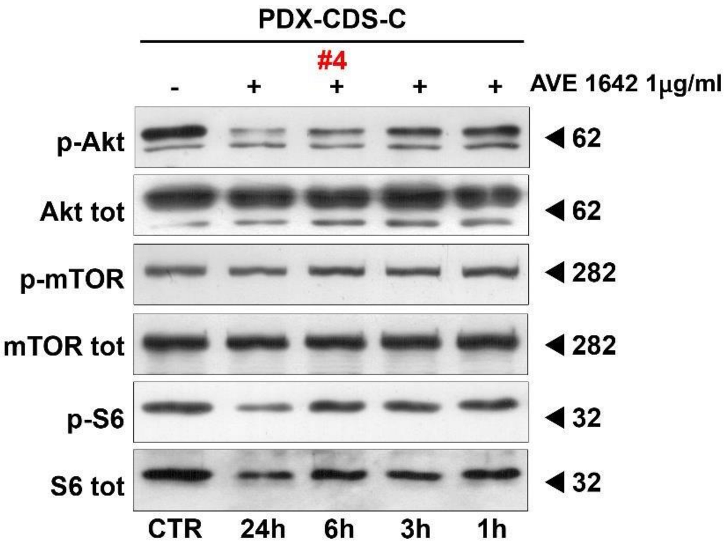

Akt signaling mediators in the PDX-CDS#4-C cells treated or not with the anti-IGF1R hAb AVE 1642

Western blotting of CDS cells treated or not with AVE 1642. Two independent experiments were performed. One representative immunoblot is shown.

Supplementary Fig. S12

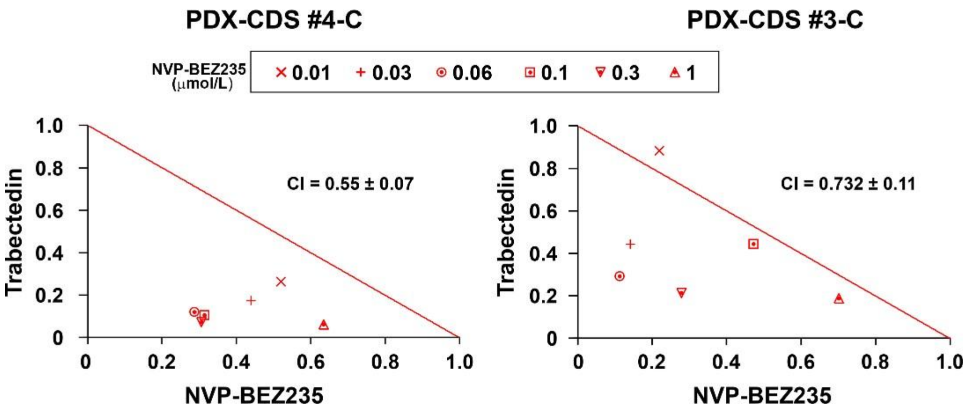

Isobologram: schematic representation of the effects of combined NVP-BEZ235 and trabectedin treatment in PDX-CDS#4-C and PDX-CDS#3-C cell lines. The CI values are represented as the mean ± SE and indicate strong synergy between the two drugs. NVP-BEZ235 concentrations are reported in the legend (µmol/L).

Supplementary Fig. S13

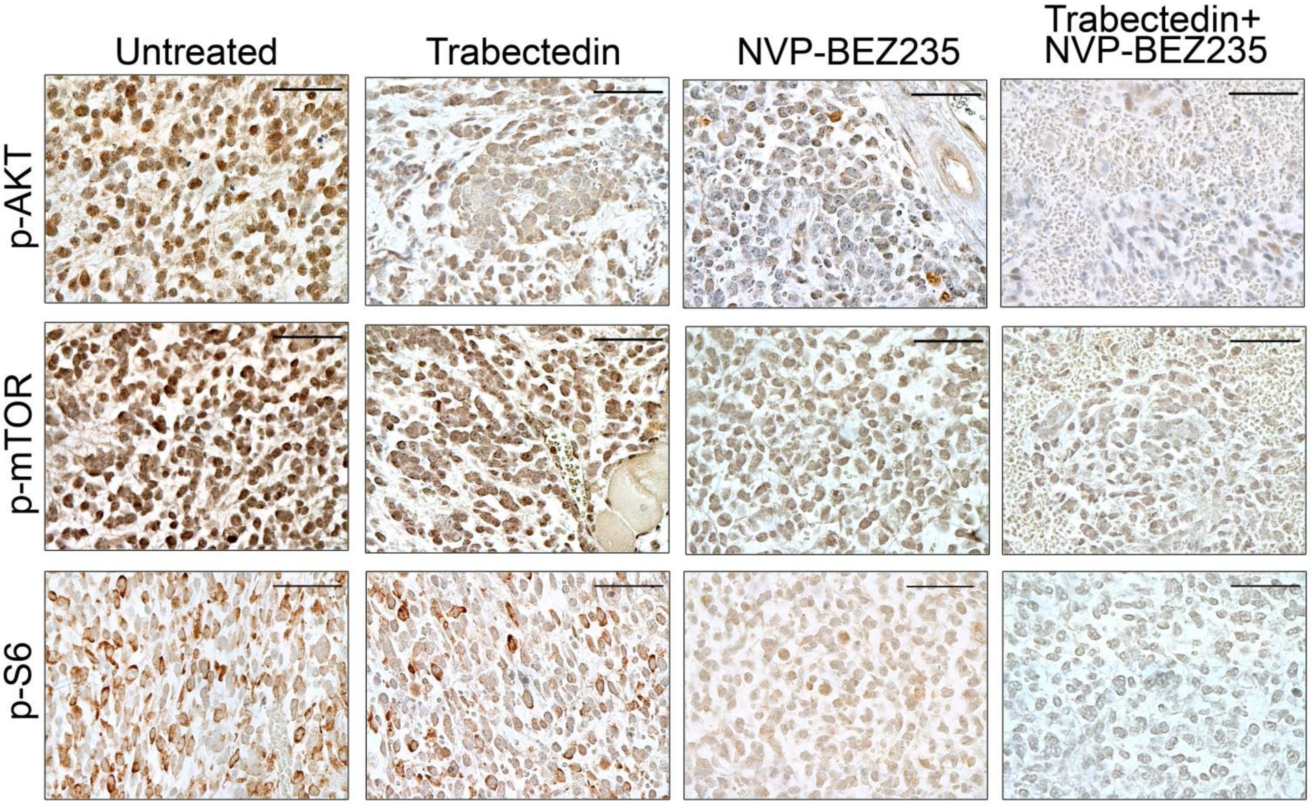

Immunohistochemical evaluation of p-Akt, p-mTOR, and p-S6 in -xenografts after s.c. injection of PDX-CDS#4-C cells treated with trabectedin, NVP-BEZ235 or the combination of the two drugs. Representative figures are shown. Bar: 100 µm.

Supplementary References

1. Dobin A, Davis CA, Schlesinger F, Drenkow J, Zaleski C, Jha S*, et al.* STAR: ultrafast universal RNA-seq aligner. Bioinformatics **2013**;29:15-21
2. Anders S, Pyl PT, Huber W. HTSeq--a Python framework to work with high-throughput sequencing data. Bioinformatics **2015**;31:166-9
3. Love MI, Huber W, Anders S. Moderated estimation of fold change and dispersion for RNA-seq data with DESeq2. Genome Biol **2014**;15:550
4. Venables WN RB. Statistics Complements to Modern Applied Statistics with S-Plus 1997.
5. Mendez M. CS, Tyrrell P.N. Using Cluster Analysis to Assess the Impact of Dataset Heterogeneity on Deep Convolutional Network Accuracy: A First Glance. 2020. 13 p.
6. Zou GY. Toward using confidence intervals to compare correlations. Psychol Methods **2007**;12:399-413
7. Bonett DG, and Thomas A. Wright. Sample Size Requirements for Estimating Pearson, Kendall and Spearman Correlations. 2000.
8. Snedecor GWaC, W.G. Statistical Methods. Iowa State University Press, Ames; 1980.
9. Gu Z, Eils R, Schlesner M. Complex heatmaps reveal patterns and correlations in multidimensional genomic data. Bioinformatics **2016**;32:2847-9
10. Subramanian A, Tamayo P, Mootha VK, Mukherjee S, Ebert BL, Gillette MA*, et al.* Gene set enrichment analysis: a knowledge-based approach for interpreting genome-wide expression profiles. Proc Natl Acad Sci U S A **2005**;102:15545-50
11. Wei T. SV. R package “corrplot”: Visualization of a Correlation Matrix. Statistician 2017. 56 p.
12. Fleming DS, Miller LC. Leading edge analysis of transcriptomic changes during pseudorabies virus infection. Genom Data **2016**;10:104-6
13. Nanni P, Landuzzi L, Manara MC, Righi A, Nicoletti G, Cristalli C*, et al.* Bone sarcoma patient-derived xenografts are faithful and stable preclinical models for molecular and therapeutic investigations. Sci Rep **2019**;9:12174
14. Garofalo C, Manara MC, Nicoletti G, Marino MT, Lollini PL, Astolfi A*, et al.* Efficacy of and resistance to anti-IGF-1R therapies in Ewing's sarcoma is dependent on insulin receptor signaling. Oncogene **2011**;30:2730-40
15. Riggi N, Suva ML, Suva D, Cironi L, Provero P, Tercier S*, et al.* EWS-FLI-1 expression triggers a Ewing's sarcoma initiation program in primary human mesenchymal stem cells. Cancer Res **2008**;68:2176-85
